# Impact of Intended Isocaloric Early versus Late Time‐Restricted Eating on Plasma Lipidome in Women with Overweight or Obesity: Secondary Analysis of the ChronoFast Trial

**DOI:** 10.1002/advs.202507149

**Published:** 2025-11-04

**Authors:** Kristof Szekely, Mathias J. Gerl, Beeke Peters, Julia Schwarz, Bettina Schuppelius, Markus Damm, Jorge R. Soliz‐Rueda, Ratika Sehgal, Michail Lazaratos, Christian Klose, Kai Simons, Andreas F. H. Pfeiffer, Annette Schürmann, Achim Kramer, Andreas Michalsen, Olga Pivovarova‐Ramich

**Affiliations:** ^1^ Department of Molecular Metabolism and Precision Nutrition German Institute of Human Nutrition Potsdam‐Rehbruecke 14558 Nuthetal Germany; ^2^ Department of Endocrinology and Metabolism Charité – Universitätsmedizin Berlin Corporate Member of Freie Universität Berlin and Humboldt‐Universität zu Berlin 10117 Berlin Germany; ^3^ German Center for Diabetes Research (DZD) 85764 München‐Neuherberg Germany; ^4^ Lipotype GmbH 01307 Dresden Germany; ^5^ Institute of Agricultural and Nutritional Sciences Martin Luther University Halle‐Wittenberg 06120 Halle Germany; ^6^ Nutrigenomics Research Group Biochemistry and Biotechnology Department Universitat Rovira i Virgili Tarragona 43007 Spain; ^7^ Department of Experimental Diabetology German Institute of Human Nutrition Potsdam‐Rehbruecke 14558 Nuthetal Germany; ^8^ Institute of Nutritional Science University of Potsdam 14558 Nuthetal Germany; ^9^ Laboratory of Chronobiology Charité – Universitätsmedizin Berlin Corporate Member of Freie Universität Berlin and Humboldt‐Universität zu Berlin 10117 Berlin Germany; ^10^ Department of Internal and Integrative Medicine Immanuel Hospital Berlin 14109 Berlin Germany; ^11^ Epidemiology and Health Economics Institute of Social Medicine Charité – Universitätsmedizin Berlin Corporate Member of Freie Universität Berlin, and Humboldt‐Universität zu Berlin 10117 Berlin Germany

**Keywords:** adipose tissue, eating timing, lipid metabolism, plasma lipidome, time‐restricted eating

## Abstract

Time‐restricted eating (TRE) is a promising strategy against metabolic disorders, but its effects on lipid metabolism remain controversial. The present research assesses and compares the impact of early (eTRE) versus late (lTRE) TRE on the plasma lipidomic profile. This is an exploratory outcome of the previously published randomized crossover trial, which examines 31 women with overweight or obesity who follow a two‐week eTRE and a two‐week lTRE in an intended isocaloric setting. Blood plasma and subcutaneous adipose tissue biopsies are analyzed using shotgun lipidomics and transcriptomics, respectively. Between interventions and within the lTRE, lipid species and classes, as well as enzyme activity indices, are not substantially changed. Within the eTRE, changes are observed for 103 lipid species, including a reduction of ceramide and phosphatidylcholine classes, and for the desaturation indices D5D, D6D, and D9D, as well as the elongation index ELOVL6. Combined analysis of plasma lipidome and adipose tissue reveals alterations in the glycerophospholipid pathway and in the expression of phospholipase enzymes *PLB1, PLA2G6*, and *PLAG4B*, dependent on TRE timing. These results suggest that eating timing during TRE may be crucial for remodeling the plasma lipidome and adipose tissue transcriptome and highlight the need of future lipidomic research in TRE.

## Graphical abstract

Women with overweight or obesity follow intended isocaloric early and late time‐restricted eating (TRE) in a crossover trial. Shotgun plasma lipidomics reveals no significant differences between interventions, but identify pronounced lipid changes—including reductions in ceramides and phosphatidylcholines—within the early TRE. Integration with adipose tissue transcriptome data reveals the impact of eating timing on the glycerophospholipid pathway.

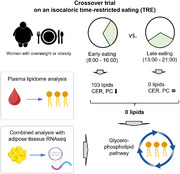

## 1 Introduction

In recent years, intermittent fasting (IF) has become increasingly popular within the scientific community and beyond. One type of IF is time‐restricted eating (TRE), in which food intake is limited to 6 to 10 h per day. The reason for the popularity of this approach, in addition to its numerous beneficial effects, is its easy implementation.^[^ ^1^ ^]^ The effects of TRE have been intensively studied in recent years, and improvements have been observed for body weight,^[^ ^2^, ^3^, ^4^, ^5^, ^6^, ^7^, ^8^, ^9^, ^10^, ^11^, ^12^, ^13^, ^14^, ^15^, ^16^, ^17^ ^]^ body fat,^[^ ^3^, ^4^, ^7^, ^8^, ^10^, ^12^, ^13^, ^15^, ^16^ ^]^ waist circumference,^[^ ^9^, ^10^, ^15^, ^18^ ^]^ inflammatory markers,^[^ ^8^, ^16^ ^]^ oxidative stress,^[^ ^7^, ^19^ ^]^ blood pressure,^[^ ^6^, ^10^, ^12^, ^19^ ^]^ blood glucose parameters,^[^ ^3^, ^7^, ^8^, ^11^, ^14^, ^19^, ^20^ ^]^ sleep, and overall quality of life.^[^ ^2^, ^9^ ^]^ Therefore, TRE is a promising prevention and treatment approach improving the metabolic state in obesity and type 2 diabetes. In these diseases, dysfunctions of carbohydrate and lipid metabolism are closely interrelated. In particular, dyslipidemia has been described in type 2 diabetes mellitus.^[^ ^21^ ^]^ However, the effects of TRE on lipid metabolism are partly controversial.

Several TRE trials have found improvements in triglyceride,^[^ ^3^, ^4^, ^8^, ^14^, ^22^ ^]^ total and low‐density lipoprotein (LDL) cholesterol levels.^[^ ^10^, ^22^ ^]^ Nevertheless, these results vary depending on the study design and study cohort, with some TRE studies showing no change or even a worsening of lipid metabolism.^[^ ^5^, ^6^, ^7^, ^9^, ^10^, ^11^, ^12^, ^13^, ^19^, ^20^, ^23^ ^]^ Further, it remains generally unclear whether shortening of the eating window provides additional metabolic effects compared to continuous caloric restriction and/or changes in dietary composition, and which timing of eating during TRE is more beneficial. Remarkably, most clinical studies suggest metabolic benefits of eTRE,^[^ ^11^, ^19^, ^20^, ^24^ ^]^ which are hypothesized to be superior compared to the lTRE.^[^ ^25^ ^]^ However, trials directly comparing eTRE and lTRE are very limited,^[^ ^14^, ^26^, ^27^, ^28^ ^]^ and their results are also inconsistent. While some trials found a decrease in triglycerides and LDL cholesterol in both interventions, showing no difference between the early and late eating windows,^[^ ^14^, ^28^ ^]^ other studies observed an aggravation of LDL cholesterol within both diets.^[^ ^27^ ^]^

The development of novel research techniques, such as high‐throughput shotgun lipidomics, offers a significantly more accurate approach to assess a broader range of plasma lipids, opening new horizons in lipid research compared to lipid parameters used in everyday clinical practice.^[^ ^29^ ^]^ In particular, the lipidome analysis can provide more insights into the mechanisms of TRE. Using this method, a reduction in sphingosines and different sphingomyelins was observed after some IF interventions, e.g., the Ramadan fast.^[^ ^30^ ^]^ In our previous research, shotgun lipidomic analysis allowed to characterize in detail the daytime‐dependent changes in postprandial lipid metabolism^[^ ^31^ ^]^ and confirmed that the timing of meal intake modulates dietary‐induced lipid responses. However, so far, no lipidomic study has directly compared the effects of early and late TRE, especially in a crossover trial. Therefore, the aim of the present research was to assess and compare the impact of isocaloric eTRE versus late lTRE on the plasma lipidomic profile. Based on previous literature, we expected that eTRE would induce more pronounced effects on plasma lipidome compared to the lTRE. The study was a secondary analysis of the crossover ChronoFast trial (NCT04351672) conducted in women with overweight or obesity, which primary outcome data were published previously.^[^ ^32^ ^]^ Because an additional aim was to assess the contribution of adipose tissue in lipid changes upon TRE, we combined the analysis of plasma lipidome with gene expression profiling in the subcutaneous adipose tissue (SAT).

## 2 Results

### 2.1 Study Population and Adherence to Dietary Interventions

Thirty‐one female subjects with overweight or obesity (age: 62 (53–65) years; body mass index (BMI): 30.5 (2.9) kg m^−2^) – 13 with impaired fasting glucose and/or impaired glucose tolerance (IFG/IGT) and 18 with normal glucose tolerance (NGT) – completed this study. 26 participants were postmenopausal, and five were premenopausal. Five participants have taken lipid‐lowering medication, which was continued during the whole study period. Baseline characteristics of study participants are shown in **Table** **1**.

**Table 1:** Baseline characteristics of study participants.

Characteristics	
N (% female)	31 (100%)
Age	62 (53–65)
BMI (kg m^−2^)	30.5 (2.9)
Weight (kg)	82.5 (8.4)
Fat mass (kg)	34.1 (6.6)
Lean mass (kg)	48.4 (5.4)
Waist circumference (cm)	99 (9)
Total cholesterol (mmol L^−1^)	5.63 (0.92)
HDL cholesterol (mmol L^−1^)	1.46 (1.33–1.65)
LDL cholesterol (mmol L^−1^)	3.47 (0.82)
Triglycerides (mmol L^−1^)	1.35 (0.62)
Fasting glucose (mg dL^−1^)	90 (87–96)
Systolic blood pressure (mmHg)	117 (110–135)
Diastolic blood pressure (mmHg)	75 (68–79)
Glycemic state (NGT:IFG/IGT)	18:13
Chronotype (early:intermediate:late)	18:11:2
HbA_1c_ (%)	5.50 (5.22–5.70)

Abbreviations: BMI, body mass index; HDL, high‐density lipoprotein; LDL, low‐density lipoprotein; NGT, normal glucose tolerance; IFG/IGT, impaired fasting glucose/impaired glucose tolerance; HbA_1c_, hemoglobin A_1C._ For parameters with normal distribution mean (standard deviation, SD) are reported, for parameters with non‐normal distribution median (25th and 75th interquartile range, IQR) are reported.

**Figure 1:**
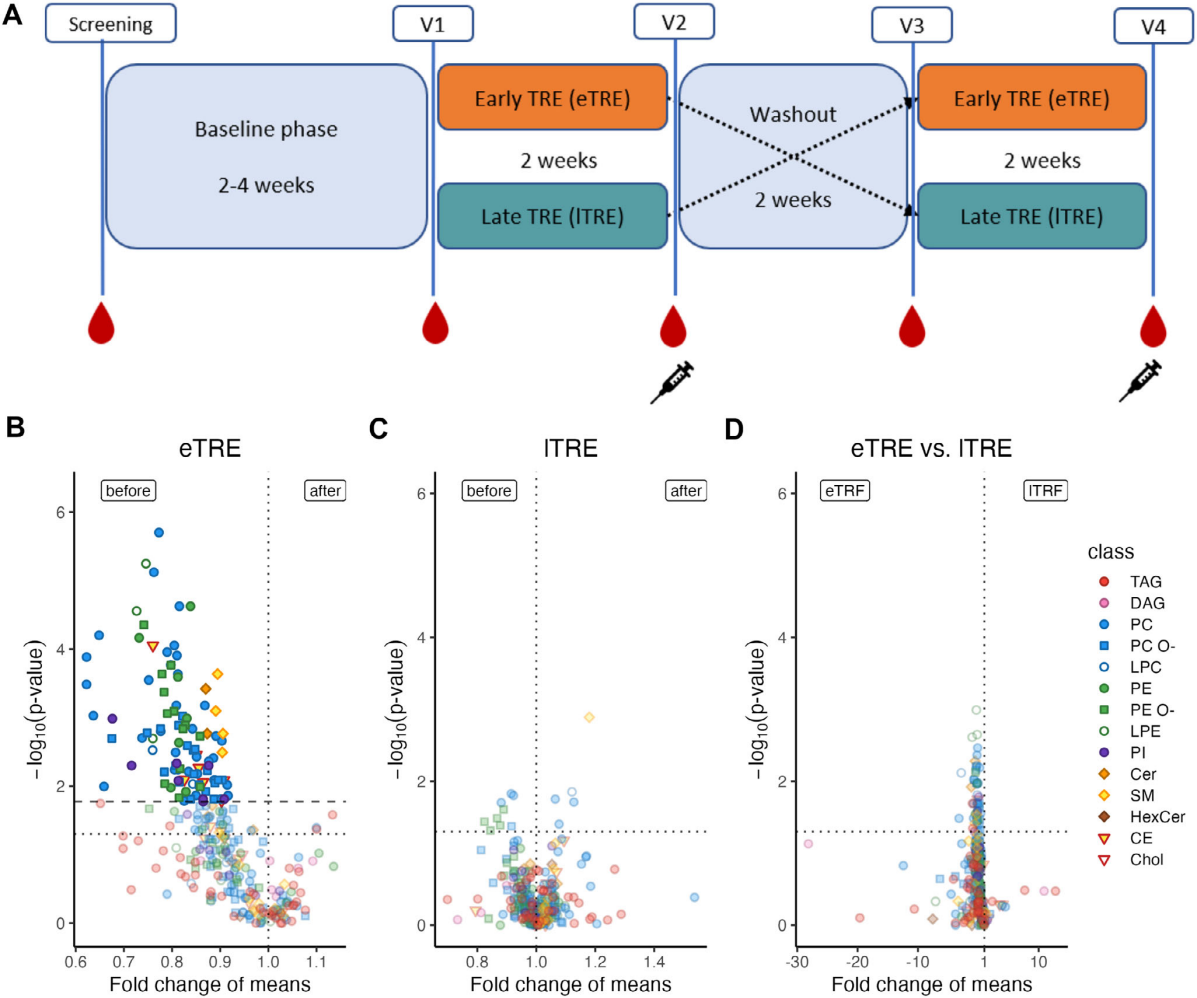
Impact of eTRE and lTRE on lipid species profiles in plasma. A) Study design. Early TRE (eTRE, orange), eating window between 8 am and 4 pm; late TRE (lTRE, petrol), eating window between 1 pm and 9 pm; red drops indicate the collection of blood samples for lipidomic analysis and syringes indicate the SAT samples; V, visit. B) Changes in lipid species profiles within the eTRE intervention. C) Changes in lipid species profiles within the lTRE intervention. D) Changes in lipid species profiles between eTRE and lTRE intervention. In (B) and (C): Volcano plots show comparisons of lipid species before versus after the intervention (N = 30 for eTRE and N = 31 for lTRE). D): Volcano plot shows comparisons of lipid species between the two interventions (eTRE vs lTRE). Only lipid species with *p* < 0.05 in the paired Mann‐Whitney U Test (before correction for multiple testing) are shown. The *y*‐axis displays the negative logarithm of *p*‐values (uncorrected), and the *x*‐axis shows fold changes of mean amounts. A dotted vertical line at a fold change of 1 indicates no change in lipid abundance. Titles indicate the intervention, and the labels at the top of each plot show the direction of fold change: “before versus after” for (B) and (C), and “eTRE versus lTRE” for (D). Lipid (sub‐)species not significant after Benjamini–Hochberg correction are shown translucent; significant species are shown opaque with an outline. In (B), the horizontal line indicates the highest *p*‐value significant after Benjamini‐Hochberg correction. In (C) and (D), no significance threshold could be established. All plots include a dotted horizontal line at *p*‐value = 0.05 (uncorrected) as a visual reference.

Details of this trial are described in **Figure** **1**A, Table S1 (Supporting Information), the Experimental Section, and Peters et al.^[^ ^32^, ^33^ ^]^ In brief, after a two‐to‐four‐week baseline period, participants followed a two‐week eTRE and a two‐week lTRE intervention in a crossover design. They showed a high adherence to the 8‐h eating window in both TRE interventions. During the baseline phase, the participants' median eating window was 11:48 (10:50–13:15) h per day, which decreased to 7:09 (6:57–7:21) h during the eTRE intervention, and to 6:57 (6:39–7:16) h during the lTRE intervention. The subjects maintained a high timely compliance of 96.5% (6.3%) in eTRE and 97.7% (6.1%) in lTRE. There was no difference in macronutrient composition and physical activity between and within the two TRE interventions (Table S2, Supporting Information). Despite intensive real‐time dietary monitoring to ensure an isocaloric intake,^[^ ^33^ ^]^ a minor, but statistically significant, decrease in daily energy intake was observed within the eTRE intervention (−167 kcal; *P* < 0.001), but not within lTRE (−97 kcal; *P* = 0.06). Minimal weight loss was observed within both eTRE (−1.08 kg; *P* < 0.001) and lTRE (−0.44 kg; *P *= 0.01), with a between‐intervention difference of 0.65 kg (*P* = 0.012). Fat mass loss (−0.61 kg, *P* = 0.002) and lean mass loss (−0.57 kg; *P *= 0.04) were found within eTRE only, but their percentage to body weight was not changed within and between both interventions (Table S3, Supporting Information). Total cholesterol, LDL cholesterol, and triglyceride concentrations were not affected by both interventions. High‐density lipoprotein (HDL) cholesterol declined within both eTRE (−0.10 mmol L^−1^; *P* < 0.001) and lTRE (−0.07 mmol L^−1^; *P* = 0.003), with no difference between the interventions. Body weight, body composition, and clinical lipid parameters were comparable at the beginning of eTRE and lTRE (Table S3, Supporting Information).

### 2.2 Detected Plasma Lipids

Lipid profiles in plasma samples could be determined in 30 subjects for the eTRE intervention and in 31 subjects for the lTRE intervention. Lipidomics analysis yielded on average 10 534 ± 2768 pmol (*n* = 154) of lipids per µL of sample. Lipid species from 14 lipid classes (cholesterol (Chol), cholesterol esters (CE), triacylglycerols (TAG), diacylglycerols (DAG), phosphatidylcholines (PCs), phosphatidylcholine ethers (PC O‐), phosphatidylethanolamines (PE), phosphatidylethanolamine ethers (PE O‐), phosphatidylinositols (PI), lysophosphatidylcholines (LPC), lysophosphatidylcholine ethers (LPC O‐), lysophosphatidylethanolamines (LPE), sphingomyelins (SM), and ceramides (CER) were identified and quantified (Table S4, Supporting Information). Lipid species present in less than 70% of all samples were excluded, resulting in a total of 300 lipid species for further analysis. This procedure accounted for 99% (98.9 ± 0.6 (*n* = 154)) of the total lipid content.

Plasma lipid patterns were analyzed for changes of i) lipid species, ii) lipid classes, iii) fatty acids; iv) indices of desaturase and elongase activities; v) double bonds; vi) total carbon lengths –compared effects of both interventions and within the eTRE and lTRE.

### 2.3 Effects of eTRE and lTRE on Plasma Lipid Species and Classes

Between eTRE and lTRE interventions, no alterations of lipid species were revealed (here and elsewhere: only changes of lipid parameters that remained significant after the correction for multiple testing, are described) (Figure 1B–D; Table S5, Supporting Information). Despite the lack of between‐group differences, we conducted an additional exploratory analysis of the within‐intervention changes. Within the eTRE, a decrease in 103 lipid species compared to the beginning of the intervention was found, while no lipid species were altered within the lTRE (Figure 1B; Table S5, Supporting Information).

**Figure 2:**
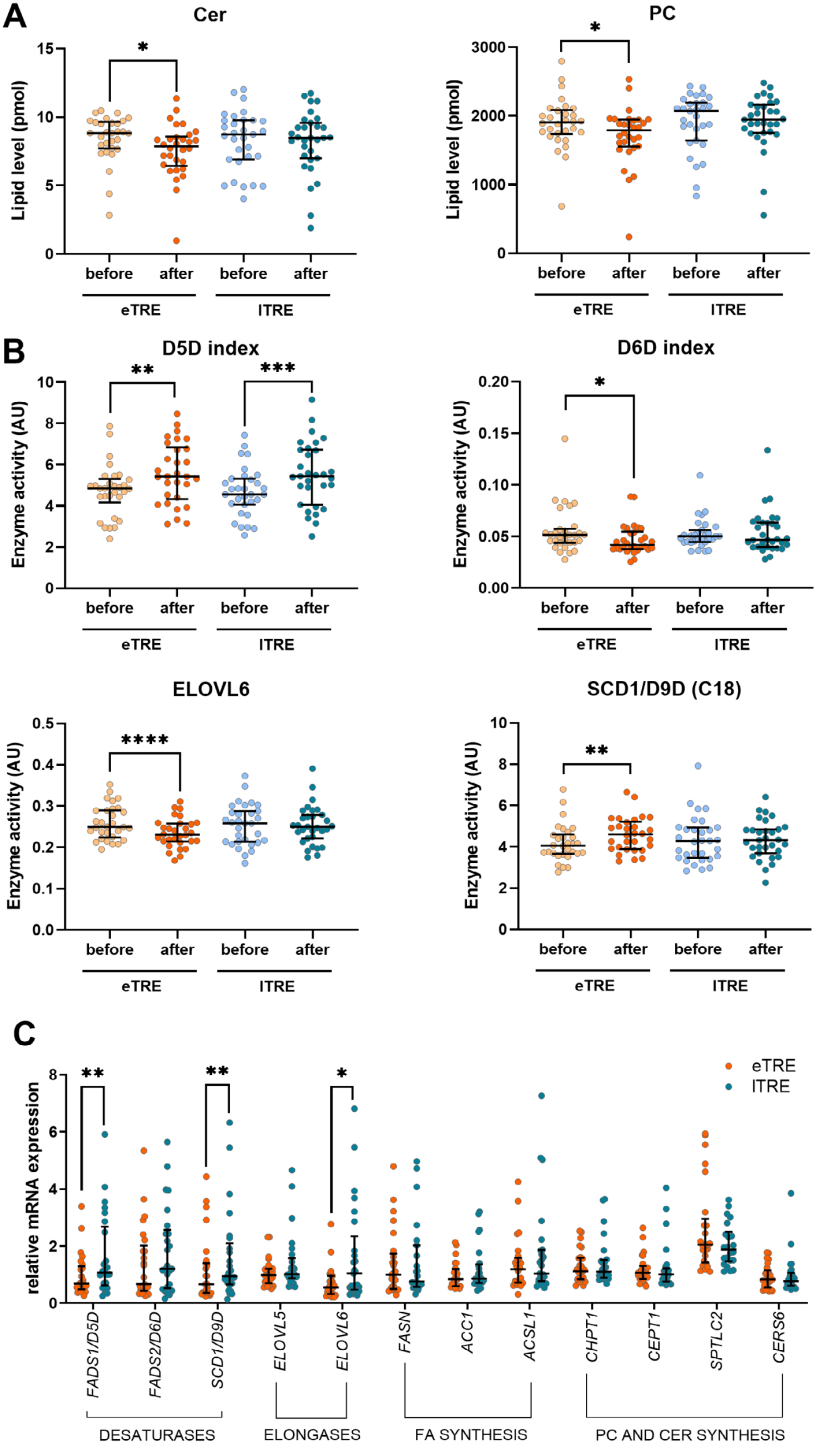
Lipid classes and enzyme activity indices in plasma and gene expression in adipose tissue. A) Ceramide (Cer) and phosphatidylcholine (PC) lipid classes demonstrated significant alterations within eTRE (lipid levels before and after the intervention are shown with beige and orange dots, respectively) or lTRE (lipid levels before and after the intervention are shown with blue and petrol dots, respectively) intervention. B) Activity indices of key lipid metabolism enzymes showed significant alterations within eTRE (beige/orange) or lTRE (blue/petrol) intervention. In (A) and (B): N = 30 for eTRE and N = 31 for lTRE; data are shown as dot plots with median and IQR. The data were analyzed using the Wilcoxon signed‐rank test. The Benjamini‐Hochberg method was used to control for false discovery rate (FDR). Only lipid indices with significant differences after adjusting for multiple testing are shown. Significance levels are encoded as follows: ^*^ *p* < 0.05, ^**^ *p* < 0.01, ^***^ *p* < 0.001. C) Expression of key lipid enzymes in subcutaneous adipose tissue (SAT). Relative mRNA expression of the desaturases *FADS1/D5D, FADS2/D6D, SCD1/D9D*; elongases *ELOVL5, ELOVL6*; FA synthesis enzymes *FASN, ACC1, ACSL1*; PC and CER synthesis enzymes *CHPT1, CEPT1, SPTLC2*, and *CERS6* in SAT samples collected after eTRE (orange dots) and lTRE (petrol dots) intervention is shown. All data were normalized to the geometric mean of the housekeeper genes *GUSB* and *RPLP0* after quantification by qPCR. N = 25; data are shown as dot plots with median and IQR. Significances are encoded as follows: ^*^ *p* < 0.05, ^**^ *p* < 0.01, ^***^ *p* < 0.001, by the Wilcoxon signed‐rank test. Abbreviations: *FADS1/D5D*, fatty acid desaturase 1/delta‐5 desaturase; *FADS2/D6D*, fatty acid desaturase 2/ delta‐6 desaturase; *SCD1/D9D*, stearoyl‐CoA desaturase/ delta‐9 desaturase; *ELOVL5*, elongation of very long chain fatty acids protein 5; *ELOVL6*, elongation of very long chain fatty acids protein 6; *FASN*, fatty acid synthase; *ACC1*, acetyl‐CoA carboxylase alpha; *ACSL1*, acyl‐CoA synthetase long chain family member 1; *CHPT1*, choline phosphotransferase 1; *CEPT1*, choline/ethanolamine phosphotransferase 1; *SPTLC2*, serine palmitoyltransferase long chain base subunit 2; *CERS6*, ceramide synthase 6; *GUSB*, glucuronidase beta; *RPLP0*, ribosomal protein lateral stalk subunit P0.

Analysis of lipid classes revealed no alterations between interventions and within the lTRE (**Figure** **2**A; Table S6, Supporting Information). Within the eTRE intervention, a reduction of CER (*P* = 0.043) and PCs (*P *= 0.043) was observed (Figure 2A; Table S6, Supporting Information).

### 2.4 Effects of eTRE and lTRE on the Elongation Indices and Lipid Carbon Chain Length

Based on the levels of specific fatty acids (FA) within complex lipids (Table S7, Supporting Information), we further quantified indices of desaturation and elongation as surrogate markers of corresponding enzyme activity (Table S8, Supporting Information). Between interventions, no differences in elongation indices elongation of very long chain fatty acids protein 5 (ELOVL5) and elongation of very long chain fatty acids protein 6 (ELOVL6) were observed (Table S8, Supporting Information), and two lipid subgroups across two lipid classes showed alterations in total carbon chain length, with each subgroup containing all lipid species from the same lipid class with identical carbon chain length (Table S10, Supporting Information). Within the eTRE, the elongation index ELOVL6 (calculated as FA 18:0 to FA 16:0 ratio^[^ ^34^ ^]^) was decreased (*P* < 0.001), whereas the ELOVL5 index (calculated as FA 20:3 to FA 18:3 ratio^[^ ^34^ ^]^) was not altered (Figure 2B; Table S9, Supporting Information). In agreement with this, the level of stearic acid 18:0 decreased within the eTRE intervention (*P* = 0.010) (Table S7, Supporting Information). The total carbon chain length was altered in 22 lipid class subgroups across ten lipid classes (Table S10, Supporting Information). Within lTRE, FA‐derived elongation indices were not altered (Table S8, Supporting Information), whereas total carbon chain length was changed in three lipid subgroups across two lipid classes (Table S10, Supporting Information).

### 2.5 Effects of eTRE and lTRE on the Desaturation Indices and Lipid Double Bond Number

Between interventions, no difference of desaturation indices delta‐5 desaturase/fatty acid desaturase 1 (D5D/FADS1), delta‐6 desaturase/fatty acid desaturase 2 (D6D/FADS2), and delta‐9 desaturase/stearoyl‐CoA desaturase (D9D/SCD1) was observed (Table S8, Supporting Information), and two lipid subgroups changed the number of double bonds (Table S11, Supporting Information). Within eTRE, the desaturation indices D5D/FADS1 (calculated as FA 20:4 to FA 20:3 ratio,^[^ ^34^, ^35^, ^36^ ^]^ *P* = 0.002) and D9D/SCD1 for C18 (calculated as FA 18:1 to FA 18:0 ratio,^[^ ^34^, ^37^, ^38^ ^]^ *P* = 0.007) increased, while the indices of D6D/FADS2 (calculated as FA 18:3 to FA 18:2 ratio,^[^ ^34^ ^]^ *P* = 0.035) decreased (Figure 2B; Table S9, Supporting Information). In agreement with this, the level of alpha‐linolenic acid 18:3, used for the calculation of the D6D index, decreased following the eTRE intervention (*P* = 0.026) (Table S7, Supporting Information). In general, the number of double bonds showed alterations in 39 lipid subgroups across 12 lipid classes, with each subgroup containing all lipid species from the same lipid class with an identical number of double bonds (Table S11, Supporting Information). Within lTRE, only the D5D/FADS1 index was increased (*P *< 0.001) (Figure 2B; Table S9, Supporting Information), and the number of double bonds showed alterations in six lipid subgroups across five lipid classes (Table S11, Supporting Information). No FA levels were changed within lTRE (Table S7, Supporting Information).

### 2.6 Effects of eTRE and lTRE on Key Lipid Enzymes in Adipose Tissue

We further hypothesized that the expression of key lipid metabolism genes in adipose tissue can be affected by eTRE and lTRE and is consistent with the alterations of ceramides and phosphatidylcholines observed in plasma. To investigate this, we assessed the expression of key genes involved in phosphatidylcholine biosynthesis (choline phosphotransferase 1 (*CHPT1), choline/ethanolamine phosphotransferase 1, (CETP1)*) and ceramide biosynthesis (serine palmitoyltransferase long chain base subunit 2 (*SPTLC2*), ceramide synthase 6 (CERS6)) in SAT samples collected after the eTRE and lTRE using qPCR (Table S13, Supporting Information). No differences of the expression of these genes were found between both interventions (Figure 2C).

In addition to these genes, we have investigated the expression of genes responsible for key lipid metabolism processes in SAT, i.e., 1) desaturases (fatty acid desaturase 1 (*FADS1* also known as *D5D*), fatty acid desaturase 1 (*FADS2* also known as *D6D*), and stearoyl‐CoA desaturase *(SCD1* also known as *D9D)*), 2) elongases (*ELOVL5* and *ELOVL6*), and 3) genes of fatty acid synthesis (fatty acid synthase (*FASN*), acetyl‐CoA carboxylase alpha (ACC1), and acyl‐CoA synthetase long chain family member 1 (*ACSL1)*). Most of the selected genes (as well as ceramide and phosphatidylcholine biosynthesis genes mentioned above) demonstrated circadian rhythms in previous human research.^[^ ^39^, ^40^, ^41^, ^42^, ^43^, ^44^ ^]^ We therefore hypothesized that the 24‐h rhythms of these lipid species can be influenced by the timing of the eating window during eTRE and lTRE interventions. The mRNA expression after the lTRE intervention was higher than after eTRE for the desaturases *FADS1* (*P* = 0.008) and *SCD1* (*P* = 0.008) as well as for the elongase *ELOVL6* (*P* = 0.013) (Figure 2D). For all other genes examined, no differences between eTRE and lTRE expression levels were found.

### 2.7 Lipid Pathway Enrichment combined with Adipose Tissue Analysis

**Figure 3:**
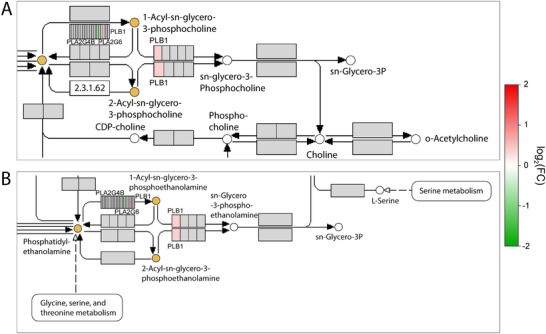
Selected areas of the glycerophospholipid pathway combining lipid and gene expression data. A) Upper zoomed area of the glycerophospholipid pathway. B) Lower zoomed area of the glycerophospholipid pathway. In (A) and (B): Glycerophospholipid pathway (KEGG ID: *hsa00564*) was identified by the lipid pathway enrichment analysis of plasma lipidome, and its full map is presented in Figure S1 (Supporting Information). The output of the lipid pathway enrichment analysis was subjected to the metaKEGG tool, and the KEGG compounds belonging to the enriched pathway were highlighted. Genes found in the SAT transcriptome dataset were directly mapped on the pathway and colored according to their log_2_(FC) translated to a color scale. In the color scale, red color means a positive log_2_(FC), i.e., the transcript was upregulated in ITRE compared to the eTRE, whereas green color means a negative log_2_(FC). This allowed to identify three genes coding phospholipase enzymes *PLB1, PLA2G6*, and *PLAG4B*, which were regulated differently after eTRE and lTRE interventions. Abbreviations: *PLB1*, phospholipase B1; *PLA2G6*, phospholipase A2 group VI; *PLAG4B*, phospholipase A2 group IVB.

To elucidate further lipid metabolism pathways that underwent remodeling by the TRE interventions, we conducted a lipid pathway enrichment analysis of the altered plasma lipidome as described in the Experimental Section. This analysis identified that the glycerophospholipid pathway was significantly affected by eTRE (Table S14, Supporting Information). Based on the SAT RNAseq dataset (GSE287198) collected in the same trial after both interventions, we have further analyzed the changes in expression profile of adipose tissue genes involved in the glycerophospholipid pathway (**Figure** **3**A). Using the metaKEGG tool [https://metakegg.apps.dzd‐ev.org, https://github.com/dife‐bioinformatics/metaKEGG], we visualized observed pathway changes on both lipidomic and the transcriptomic layers. Genes found in the RNAseq dataset were directly mapped on the pathway and colored according to their log_2_(FC) (Figure S1, Supporting Information). This allowed to identify three genes, involved in the glycerophospholipid pathway and coding phospholipase enzymes, which were regulated differently after eTRE and lTRE interventions (Figure 3B,C). Expression of phospholipase B1 (*PLB1)* and phospholipase A2 group VI (*PLA2G6)* were downregulated, while phospholipase A2 group IVB (*PLA2G4B)* was upregulated after the eTRE intervention compared to the lTRE.

## 3 Discussion

In this work, we assessed and compared, for the first time, effects of eTRE and lTRE regimens on the human plasma lipidome using high throughput lipidomic analysis. Importantly, the TRE interventions were intended to be isocaloric, and study adherence^[^ ^33^ ^]^ was carefully controlled to ensure that plasma lipid changes were caused by the shortening and/or timing of the eating window itself, independent of calorie deficit, changes in food composition, or physical activity. We achieved high timely adherence to both interventions, unchanged dietary composition and physical activity, although minimal caloric reduction could not be completely avoided within eTRE.

The main finding of this study is that no differences in effects on plasma lipidome were found between eTRE and lTRE, considering lipid species and classes, enzyme activity indices, carbon chain length, and the number of double bonds. This aligns with previously published studies that have found no difference in the impact of eTRE and lTRE on clinical lipid metabolism parameters, such as cholesterol and triglycerides, when directly comparing both interventions.^[^ ^14^, ^27^, ^28^ ^]^ To date, only one study by Hutchison et al. has compared eTRE and lTRE in a crossover design,^[^ ^14^ ^]^ finding that fasting triglycerides and postprandial glucose decreased similarly with both diets. Another parallel‐arm trial showed comparable beneficial effects of early, late, and self‐selected TRE combined with a Mediterranean diet compared to a Mediterranean diet alone on blood lipids as well as visceral adipose tissue volume in individuals with overweight or obesity.^[^ ^28^ ^]^ A recent meta‐analysis also confirmed comparable results of eTRE versus lTRE on clinical lipid metabolism parameters.^[^ ^45^ ^]^ Further, our lipidomics data are consistent with data on blood lipids, glycemic control, and other cardiometabolic parameters from the same ChronoFast trial, which demonstrated no clinically relevant improvents between and within interventions in a nearly isocaloric setting.^[^ ^33^ ^]^

Nevertheless, despite the lack of between‐group comparisons, we cautiously interpreted and discussed the within‐intervention data, considering them as exploratory and hypothesis‐generating. In within‐intervention comparisons, eTRE had more pronounced effects on the plasma lipidome compared to lTRE. Indeed, eTRE caused a decrease in 103 of the analyzed lipid species compared to the levels before intervention, whereas lTRE showed no effect on lipid species. Similar differences were also observed for lipid classes and enzyme activity indices. Specifically, eTRE caused a reduction of ceramides and phosphatidylcholines, as well as alterations of several elongation and desaturation indices, while lTRE caused a change in the D5D index only. Accordingly, eTRE induced more changes in the number of total double bonds and total carbon chain length per class than lTRE.

The marked within‐intervention impact of eTRE on the plasma lipidome aligns with published data showing superior metabolic effects of eTRE compared to lTRE.^[^ ^11^, ^19^, ^20^, ^24^ ^]^ Numerous eTRE studies have shown that restricting food intake to the beginning of the day enhances insulin sensitivity, beta‐cell responsiveness, and reduces blood pressure, inflammation, and oxidative stress.^[^ ^11^, ^19^, ^20^, ^24^ ^]^ Conversely, lTRE, where food intake is restricted to the late afternoon or evening (after 4 pm), has either shown no effect or worsened blood glucose, beta‐cell responsiveness, and lipid levels.^[^ ^5^, ^7^, ^8^, ^13^, ^46^ ^]^ The superior metabolic effects of eTRE compared to the lTRE may be explained by circadian rhythms of key metabolic processes, including lipid metabolism.^[^ ^25^, ^47^ ^]^

Indeed, the molecular clock machinery induces circadian rhythms of essential lipid metabolism enzymes via clock‐controlled transcription factors such as PPARs, PGC‐1alpha, and SREBPs.^[^ ^48^, ^49^, ^50^, ^51^ ^]^ Correspondingly, a large part of the plasma lipidome demonstrates circadian rhythmicity, as demonstrated in constant routine studies, confirming the essential role of the endogenous circadian clock in lipid homeostasis.^[^ ^49^, ^52^, ^53^ ^]^ Notably, circadian lipidome organization is altered both in circulation and in the adipose tissue in subjects with type 2 diabetes, suggesting a strong link between lipid rhythm dysregulation and diabetes pathogenesis.^[^ ^54^ ^]^ Further, we previously found that the plasma lipidome responds differently to the same meal depending on the time of day, with approximately one‐third of all lipids showing greater postprandial changes after a morning meal compared to an afternoon meal.^[^ ^31^ ^]^ This observation aligns with our finding that eTRE has more pronounced within‐intervention effects on the plasma lipidome compared to lTRE.

Results of our study are also, in some degree, comparable with data of Madkur et al.,^[^ ^30^ ^]^ which is the only published study to date investigating the impact of any form of IF – in their case, Ramadan fasting – on the plasma lipidome using lipidomic analysis. Although Ramadan fasting and the TRE interventions studied in our trial differ in their eating timing, the two studies remain quite comparable as different forms of IF. The study reported similar lipid pattern changes, particularly for sphingomyelins, after 29–30 days of the Ramadan fasting, but in contrast to our data, the ceramide class did not change.

Our finding on ceramide reduction by eTRE may be clinically relevant, as elevated ceramide levels are associated with metabolic disfunction. Numerous studies have described increased ceramide levels in context of type 2 diabetes and cardiovascular diseases,^[^ ^55^, ^56^, ^57^, ^58^, ^59^ ^]^ as well as obesity and weight gain.^[^ ^60^, ^61^ ^]^ Ceramides are even being explored as biomarkers for cardiovascular diseases in daily clinical practice,^[^ ^55^, ^62^, ^63^, ^64^, ^65^ ^]^ and some clinics have begun using ceramide‐based scores to identify patients at risk for cardiometabolic diseases.^[^ ^66^ ^]^ Given these findings, the observed reduction in both the total ceramide class and the single ceramide species suggests that eTRE potentially induces greater cardiometabolic benefits than lTRE. Our data obtained in the lipid enrichment analysis of the plasma lipidome support this notion. In this analysis, we revealed a number of lipid species belonging to the glycerophospholipid pathway, which has been linked to cardiovascular risk and coronary artery disease progression.^[^ ^67^, ^68^ ^]^

For estimated lipid metabolism enzyme activities, similar tendencies regarding the effects of eTRE and lTRE were observed. Specifically, in the context of diabetes, a reduction in D5D activity and an elevation in D6D activity are often described as associated factors.^[^ ^69^, ^70^, ^71^, ^72^, ^73^ ^]^ Beyond diabetes, these changes in desaturase activities are associated with metabolic syndrome, elevated fasting plasma triglycerides, C‐reactive protein, HOMA‐IR, and HbA1c.^[^ ^72^, ^74^, ^75^, ^76^, ^77^ ^]^ In this context, the observed increase in the FA‐derived D5D index in both eTRE and lTRE, along with the reduction in the D6D index in lTRE, align with published beneficial metabolic effects of TRE. However, the D9D index, which was increased within eTRE, was described to be positively associated with BMI and triglyceride levels.^[^ ^71^ ^]^ Therefore, effects of TRE timing on the lipid metabolism enzyme activities warrant careful interpretation, especially considering that the index calculation in our work was based on FAs within complex lipids and not on the free FAs as in previously published studies.

In addition to the altered elongase and desaturase indices, we found that the expression of key lipid metabolism enzymes in adipose tissue differ between the two interventions depending on eating timing, highlighting the possible contribution of adipose tissue to the TRE‐induced lipid changes in the circulation. The results of the expression analysis of the same genes in the SAT partly disagree with the enzyme indices findings in plasma. For example, in SAT, we observed higher expression of the desaturases *FADS1/D5D* and *SCD1/D9D* after the lTRE intervention compared to the eTRE, whereas no difference was detected in the corresponding enzyme indices in plasma. In turn, higher *ELOVL6* expression levels in SAT after lTRE did not align with ELOVL6 index changes in plasma.

Following observed changes in plasma ceramide and phosphatidylcholines, we also quantified the mRNA expression of key enzymes involved in their metabolism. In particular, CHPT1 and SPTLC2, essential players of ceramide and phosphatidylcholine metabolism, respectively, have been previously characterized as circadian enzymes in human muscles.^[^ ^78^ ^]^ Based on the lipidomic data and considering the *zeitgeber* role of food intake for central peripheral circadian clocks as shown in animal^[^ ^79^ ^]^ and few human trials,^[^ ^33^, ^80^, ^81^ ^]^ we expected to observe expression differences between eTRE and lTRE. However, contrary to our expectations, no significant differences were found in the expression of these and other analyzed genes between samples collected after eTRE and lTRE. This discrepancy, along with previously mentioned differences between circulating lipids and adipose tissue expression levels, may be attributable to the fact that circulating lipids are primarily synthesized in the liver; thus, their levels may not accurately reflect ongoing metabolism in SAT. Further, mRNA levels do not account for the translational and post‐translational regulation of corresponding enzymes. Similarly, we found no differences between eTRE and lTRE in the expression of genes involved in fatty acid synthesis (*FASN, ACC1*, and *ACSL1*), which have previously been shown to exhibit circadian rhythmicity.^[^ ^43^, ^44^ ^]^

By combining lipid pathway enrichment analysis and transcriptome data from SAT using the novel metaKEGG tool, we identified three genes – *PLB1, PLA2G6*, and *PLAG4B* – that appear to be involved in the effects of TRE timing on lipid homeostasis. These genes contribute to the glycerophospholipid pathway and encode phospholipase enzymes, cleaving acyl chains from the *sn‐*1 and *sn*‐2 positions of a phospholipid. Importantly, these three genes were regulated differently by the TRE timing: *PLB1* and *PLA2G6* were downregulated, while *PLAG4B* was upregulated after the eTRE intervention compared to the lTRE. Whereas PLA2G6 is known to be a proinflammatory mediator required for monocyte chemotaxis and potentially contributory to both atherosclerosis and diabetes,^[^ ^82^ ^]^ the link between both PLAG4B & PLB1 and obesity, diabetes, or cardiovascular diseases is currently unknown. Nevertheless, our findings provide new insight into the role of phospholipases in the effects of TRE.

Several strengths and limitations should be considered when interpreting the results of this work. As mentioned above, the primary strength is an absolute novelty of the data presented regarding the TRE effects on the plasma lipidome. Second, this study directly compared the effects of predefined early versus late eating windows on the plasma lipidome within a crossover design, that minimizes the impact of interindividual variability. The third strength was the maintenance of unchanged food composition, achieved through intensive nutritional counseling and careful control of food intake, as detailed previously.^[^ ^32^ ^]^ Finally, the combined analysis of the plasma lipidome and adipose tissue transcriptome represents an important strength, providing insight into the role of adipose tissue in lipid homeostasis regulation by the TRE intervention.

The important limitation of the data interpretation is an analysis of the within‐intervention changes despite the lack of between‐intervention difference. To notice, this does not automatically mean that neither intervention work. The reason can be the relatively small effect size or insufficient power, because our study was powered for assessing the primary outcome insulin sensitivity. Remarkably, we consider our data as exploratory and hypothesis‐generating, and therefore future, adequately powered lipidomic studies are needed to confirm these findings.

Another practical limitation stems from the challenges in maintaining strict caloric equivalence across the interventions. Despite intensive real‐time dietary supervision, in the eTRE, a minimal caloric reduction could not be entirely avoided, although it was lower than observed in comparable TRE studies.^[^ ^6^, ^7^, ^10^, ^27^, ^83^ ^]^ In both interventions, minor weight loss of ≈1% was observed after two weeks, consistent with previous isocaloric or nearly isocaloric TRE trials.^[^ ^14^, ^19^, ^84^, ^85^ ^]^ Of note, weight loss was slightly, but significantly, greater in the eTRE compared to the lTRE. Such minor weight changes would expectedly not meaningfully affect lipid profile, because metabolic changes are usually induced by >5% weight loss. Nevertheless, we cannot completely exclude the impact of the weight loss on lipidomic changes attributed to TRE timing, which adds complexity to the data interpretation. The third limitation lies in the indirect assessment of enzyme activity levels, which were estimated based on fatty acid levels within complex lipids and should therefore be interpreted with caution. The fourth limitation is that the trial was conducted in mostly postmenopausal women, potentially limiting the generalizability of our findings to the broader population. Finally, the intervention periods were short (two weeks), which was necessary to maximize adherence to prescribed eating times and isocaloric energy intake, facilitated by real‐time monitoring and individual counseling, and to ensure extensive documentation of interstitial glucose, physical activity, and sleep behavior.^[^ ^33^ ^]^ The limited sample size is also acknowledged as a potential limitation; however, its impact is mitigated by the crossover study design. Nevertheless, future research in other population groups and larger cohorts needs to confirm the results of our study.

In summary, our study provides new insights into the effects of TRE on plasma lipidome. Between interventions, lipid species and classes, as well as enzyme activity indices, are not substantially changed. However, the exploratory analysis of within‐intervention comparisons highlighted marked differences between the effects of eTRE and lTRE on the plasma lipidome and adipose tissue. These results suggest that eating timing during TRE might be essential for remodeling of the plasma lipidome and adipose tissue transcriptome, highlighting the need of future lipidomic research in TRE.

## 4 Experimental Section

### Study Design and Participant Characteristics

Thirty one women with overweight or obesity completed the randomized 10‐weeks crossover clinical trial, which was conducted at the German Institute of Human Nutrition Potsdam‐Rehbruecke, Germany, from April 2020 to December 2021. Details of the participant recruitment, clinical characteristics of the study participants, dietary interventions, and adherence assessment were recently published.^[^ ^32^, ^33^ ^]^ Inclusion and exclusion criteria are described in Table S1 (Supporting Information). In brief, inclusion criteria were female sex, age between 18 and 70 years, and BMI between 25 and 35 kg m^−2^. Exclusion criteria were diabetes type 1 or type 2, shift work, and travel across more than one time zone one moths before or during the study. The study protocol and informed consent form were approved by the Medical Ethics Committee of the University of Potsdam (EA No. 8/2019) and comply with the Declaration of Helsinki of 1975 as revised in 2013. All participants provided written informed consent prior to study participation. The study was registered at clinicaltrials.gov on 17.04.2020 (Identifier: NCT04351672). Study results were reported using the CONSORT 2025 checklist guideline for reporting randomized trials.^[^ ^86^ ^]^ All resources and reagents related to this trial are listed in the supporting information (Table S15, Supporting Information).

For a detailed description of the study protocol, refer to.^[^ ^32^ ^]^ In brief, after a two‐to‐four‐week baseline phase, two intended isocaloric two‐week interventions – i) eTRE and ii) lTRE – were conducted, separated by a two‐week washout phase (Figure 1A). During the baseline phase, the participants consumed their usual food quality and quantity at their usual meal times. In the eTRE intervention, participants were asked to consume the same kind and amount of food as during the baseline phase but to reduce their eating window to 8 h per day for a daytime, from 8 am to 4 pm. In the lTRE intervention, the 8‐h eating window shifted to 1 pm to 9 pm. Between the two interventions, participants returned to their usual eating times. Throughout the 8–10 weeks of the study, participants were asked to maintain their usual lifestyle habits in terms of physical activity and sleep. Eating behavior (timing, kind, and amount of food) was monitored using digital or paper‐based food records, while physical activity and sleep‐wake rhythms were controlled by the actigraphy (ActiGraph wGT3X‐BT, ActiGraph, Netherlands) and sleep logs, respectively, for 14 consecutive days of the baseline phase and during both TRE interventions. Energy intake, macronutrient composition, and eating times were assessed using the FDDB food database (Fddb Internetportale GmbH, https://fddb.info/) as described.^[^ ^32^ ^]^ All days at which the subjects followed the required TRE eating time frame with a deviation of maximally ± 30 min were considered as compliant. As a measure of physical activity, the metabolic equivalent of task (MET) was analyzed using an ActiLife software version 6.13.4 (ActiGraph, Netherlands).

At initial screening and before and after each intervention anthropometric measurements were conducted. Body composition was examined in a fasting state with a bioimpedance analyzer (BIA; Quantum S, Akern, Florence, Italy) using the BIA‐related Bodygram software (Akern, Italy). Height measurements were assessed using a stadiometer, and weight measures were assessed using a digital scale, for the calculation of BMI, defined as weight in kilograms divided by the square of the height in meters. Circumferences of the waist and hip were measured with a measuring tape.

Beyond that, metabolic examination of participants was conducted after an overnight fast, using 75 g‐oral glucose tolerance test (OGTT) and blood sample collection as described.^[^ ^32^ ^]^ Screening OGTT was used to characterize participants' metabolic state as follows: individuals were classified as having IFG if their fasting glucose was 100–125 mg dL^−1^, and as having IGT if their 2 h glucose was 140–199 mg dL^−1^.

Blood samples were collected in serum monovettes for blood glucose, HbA1c, and total lipid measurements. Participants’ chronotypes were determined using the Munich Chronotype Questionnaire (MCTQ) and the Horne–Östberg Morningness–Eveningness Questionnaire (MEQ) as described.^[^ ^32^, ^33^ ^]^ Chronotype classification in the analysis was performed on MSFsc value (midsleep on free days corrected for the sleep debt over the working days) from MCTQ using the following cut‐off‐points: MCFsc < 4 was defined as an early chronotype, MCF‐Sc >5 as a late chronotype, and intermediate values as an intermediate chronotype. Classification in the MEQ was performed as follows: a range of 59–69 defined early chronotypes, a range of 31–41 defined late chronotypes, and intermediate values for intermediate chronotypes. In case of heterogeneous results in both questionnaires, the MCTQ was used for the final chronotype classification.

### Blood Plasma and Adipose Tissue Collection

For lipidomics analysis, fasting blood samples were drawn at 9.30 a.m. before and after eTRE and lTRE interventions (visits 1–4 at Figure 1A) using EDTA monovettes (Sarstedt, Germany). Blood plasma was frozen immediately after the centrifugation and stored at −80 °C until analysis. SAT was collected periumbilically at 9.30 a.m. after both interventions (visits 2 and 4). Before each biopsy, a skin area of ≈2 × 2 cm was treated with lidocaine for anesthesia. A 3‐mm incision was then made in the skin using a fine needle (diameter 2.1 mm) connected to a vacuum syringe containing sterile NaCl solution. The resulting negative pressure allowed small pieces of tissue to be removed. These were immediately cleaned in a NaCl solution, frozen in aliquots a 0.4 g in liquid nitrogen and stored at −80 °C until analysis.

### Lipidomics Analyses of Plasma Samples

Lipidomic measurements in blood plasma were performed using mass spectrometry‐based shotgun lipidomic analysis at Lipotype GmbH (Dresden, Germany) as described.^[^ ^29^ ^]^ For lipid extraction, an equivalent of 1 µL of undiluted plasma was used, and plasma lipids were extracted with methyl tert‐butyl ether/methanol (7:2, V:V).^[^ ^87^ ^]^ Samples were analyzed by direct infusion in a QExactive mass spectrometer (Thermo Scientific, Bremen, Germany) equipped with a TriVersa NanoMate ion source (Advion Biosciences, Ithaca, New York, United States of America). Samples were analyzed in both positive and negative ion modes with a resolution of *R*_m/z = 200_ = 280 000 for MS and _Rm/z = 200_ = 17 500 for MSMS experiments in a single acquisition. Lipids were identified and quantified employing the proprietary LipotypeXplorer software. Lipid intensities were normalized to lipid class‐specific internal standards, and data were reported as molar amounts. Analytical quality was evaluated by incorporating reference and blank samples. Data were corrected for batch effects and drift based on reference samples. Lipid species present in less than 70% of all samples were excluded. The Lipidomics Standard Initiative minimal reporting checklist^[^ ^88^ ^]^ for this study can be found at https://doi.org/10.5281/zenodo.15183498.

Lipid species were annotated based on their molecular composition, indicating the sum of carbon atoms in the hydrocarbon moiety, the sum of double bonds, and the sum of hydroxyl groups. For instance, PI 34:1;0 denotes phosphatidylinositol with a total length of its fatty acids equal to 34 carbon atoms, total number of double bonds in its fatty acids equal to 1, and 0 hydroxylations. The annotation of lipid subspecies provides additional information on the exact identity of their acyl moieties and their sn‐position (if available). For example, PI 18:1;0_16:0;0 denotes phosphatidylinositol with octadecenoic (18:1;0) and hexadecanoic (16:0;0) fatty acids, for which the exact position (sn‐1 or sn‐2) in relation to the glycerol backbone cannot be discriminated (underscore “_” separating the acyl chains). In contrast, PC O‐18:1;0/16:0;0 denotes an ether‐phosphatidylcholine, where an alkyl chain with 18 carbon atoms and 1 double bond (O‐18:1;0) is ether‐bound to sn‐1 position of the glycerol, and a hexadecanoic acid (16:0;0) is connected via an ester bond to the sn‐2 position of the glycerol (slash “/” separating the chains signifies that the sn‐position on the glycerol can be resolved). Lipid identifiers from the SwissLipids database^[^ ^89^ ^]^ and the shorthand notation for MS‐derived lipid structures^[^ ^90^ ^]^ are provided. All lipid species and lipid classes identified by this method are shown in Table S3 (Supporting Information).

### Assessment of Lipid Enzyme Activity Indices

Based on the level of specific lipids, the overall amount of FA within the complex lipid was assessed. Enzyme activity indices were calculated as a ratio between two FAs and used as surrogate activity markers of corresponding enzyme responsible for the conversion from one FA to another. Following indices were assessed in this study: D5D/FADS1, D6D/FADS2,^[^ ^34^ ^]^ ELOVL5,^[^ ^34^ ^]^ ELOVL6,^[^ ^34^ ^]^ SCD1/D9D (C16), SCD1/D9D (C18), SCD1/D9D (C18+16))^[^ ^36^, ^37^, ^38^ ^]^ (Table S4, Supporting Information).

### Gene Expression Analyses of Adipose Tissue

Total RNA was isolated from SAT samples using the RNeasy Lipid Tissue Mini Kit (Qiagen, Germany). RNA concentration was determined utilizing an ND‐1000 spectrophotometer (Nanodrop, PeqLab), and the RNA quality was determined by the Bioanalyzer (Agilent Technologies, USA). Extracted RNA samples all had RIN values of ≥ 8. For the qPCR analysis, single‐stranded cDNA was generated using the QuantiTect Reverse Transcription Kit (Qiagen, Germany). Quantitative PCR (qPCR) was conducted on the ViiA 7 sequence detection system using Power SYBR Green PCR Master Mix (Applied Biosystems, USA) and specific primers (Table S5, Supporting Information). Gene expression was evaluated using the standard curve method and normalized to the geometric mean of housekeeper genes beta‐glucuronidase (*GUSB*) and P0 protein of 60S ribosomal protein large subunit (*RPLPO*). Whole‐genome transcriptome analysis was performed by RNA sequencing by BGI Genomics (BGI‐Copenhagen, Denmark) using BGISEQ platform with 100 bp paired‐end reads. RNAseq data were submitted to the GEO database with an assession number GSE287198.

### RNAseq Data Processing

RNAseq data were delivered by BGI in form of adapter and low‐quality reads cleaned sequencing data. FastQC v0.12.1 was employed to assess quality of the samples. Reads were aligned to the reference genome (GRCh38.110/hg38) using STAR 2.7.11a, and fragments per kilobase per million (FPKM) values for transcripts were determined by STRINGTIE v2.2.1, both with default options for paired reads. Differential gene expression analysis was performed via the R‐package DESeq2 (v1.34.0) pipeline. Transcripts with mean expression values > 1 FPKM, and *p* < 0.05 were considered statistically significant. RNAseq data processing was performed with R v4.1.2.

### Lipid Pathway Enrichment Combined with SAT Transcriptome Analysis

All the differentially abundant lipid species were subjected to lipid pathway enrichment analysis using online tool LIPEA (https://hyperlipea.org/). The lipid species belonging to the same lipid class were merged and KEGG pathway enrichment analysis^[^ ^91^, ^92^, ^93^, ^94^, ^95^ ^]^ output included the lipid/compound ID mapping to the enriched pathways.

The output of the lipid pathway enrichment analysis was subjected to metaKEGG tool [https://metakegg.apps.dzd‐ev.org, https://github.com/dife‐bioinformatics/metaKEGG] to visualize lipidomic and transcriptomic layers. For this application, the analysis pipeline “Bulk RNAseq mapping” was used in order to directly map the KEGG pathway that emerged from the lipid pathway enrichment analysis, bypassing the need for a gene pathway enrichment analysis. The pathway “Glycerophospholipid metabolism” (KEGG ID: *hsa00564*) was queried, using log_2_(FC) values from only significantly (1 FPKM and *p* < 0.05) differentially expressed genes of the comparison of eTRE versus lTRE. The internal KEGG compound IDs for lipid species identified in enriched pathways were provided as an additional input parameter for the metaKEGG visualization pipeline. Genes found in the SAT RNAseq dataset were directly mapped on the pathway, colored according to their log_2_(FC) translated to a color scale, while KEGG compounds were assigned a single color to highlighted their presence in the pathway.

### Statistical Analysis

Statistical power of the trial was calculated for the main study outcome, insulin sensitivity, as published previously.^[^ ^32^ ^]^ For this secondary analysis previously published nutritional lipidomic studies were referred with a similar or even lower sample size and crossover design.^[^ ^96^, ^97^ ^]^ Statistical analyses were performed with SPSS v.25 (SPSS, Chicago, IL) and R version 4.2.3 (2023‐03‐15) using tidyverse packages (version 2.0.0). *P* < 0.05 was considered statistically significant in all analyses. Comparison of lipid data between the two groups was conducted using a paired Mann‐Whitney U Test (Wilcoxon signed‐rank test). For the multiple testing correction, the Benjamini–Hochberg (BH) method was used. Data are presented as means (standard deviation, SD) for parameters with normal distribution and medians (25th and 75th interquartile range, IQR) for parameters with non‐normal distribution if not stated otherwise. Changes within eTRE or lTRE intervention (calculated as a delta of the value after the intervention minus the value before the intervention) and between‐intervention differences (calculated as a delta of the change within lTRE minus the change within eTRE) were expressed as mean (95% confidence interval, CI).

## Conflict of Interest

The authors declare no conflict of interest.

## Author Contributions

M.J.G. performed lipidomics experiments; M.J.G. and S.K. performed the data analyses; A.M., A.F.H.P., A.K., and O. P‐R conceptualized the clinical trial; B.P., J.S., and B.S. conducted the trial and provided the samples; C.K. and K.Si contributed to the lipidomics conceptualization; M.L. and R.S. conducted RNAseq data processing and lipid pathway enrichment analysis; K.S. drafted the manuscript; O.P‐R. edited the manuscript. All authors read and approved the final manuscript.

## Supporting information



Supporting Information

## Data Availability

The data that support the findings of this study are available from the corresponding author upon reasonable request.
