# Supplementary material for: Impact of Intended Isocaloric Early versus Late Time‐Restricted Eating on Plasma Lipidome in Women with Overweight or Obesity: Secondary Analysis of the ChronoFast Trial
Source: Adv Sci (Weinh). 2025 Nov 4;13(4):e07149. doi: 10.1002/advs.202507149 (PMC12822420; doi:10.1002/advs.202507149)
Supplement: Supplementary file 1 — Supporting Information [file ADVS-13-e07149-s001.pdf]

Supporting Information

Impact of Intended Isocaloric Early vs. Late Time-Restricted Eating on Plasma Lipidome in Women with Overweight or Obesity: Secondary Analysis of the ChronoFast Trial

*Kristof Szekely, Mathias J. Gerl, Beeke Peters, Julia Schwarz, Bettina Schuppelius, Markus Damm, Jorge R. Soliz-Rueda, Ratika Sehgal, Michail Lazaratos, Christian Klose, Kai Simons, Andreas F. H. Pfeiffer, Annette Schürmann, Achim Kramer, Andreas Michalsen, Olga Pivovarova-Ramich**

Table S1. Inclusion and exclusion criteria.

**Inclusion criteria**	Women<br>Age 18–70 years<br>BMI 25–35 kg/m^2
**Exclusion criteria**	Shift work<br>Travel across more than one time zone one month before a study or during the study period<br>Weight changes of more than 5% in the last 3 months<br>Pregnancy or breastfeeding<br>Severe intestinal disease, bariatric surgery in the past<br>Food allergies (individual inclusion possible after consultation with the doctor)<br>Current special diets<br>Poor quality of sleep (PSQI score < 10)<br>Diabetes type 1 or type 2<br>Severe kidney disease<br>Severe liver disease<br>Cardiac infarction or stroke in the last 6 months<br>Cancer cases in the last 2 years<br>Glucocorticoid therapy (oral)<br>Coagulation disorders<br>Taking anticoagulant medication (inclusion if pausing medication is possible)<br>Severe anaemia<br>Systemic infections<br>Severe psychiatric illnesses, addictions or depression<br>Other diseases/surgeries/medications affecting glucose metabolism, appetite or immune function

Table S2. Eating timing, food composition, and physical activity during study periods.

	**Baseline**	**eTRE**	**lTRE**
Eating window duration	11:48 (10:50 – 13:15)	07:10 (6:53 – 7:30)	07:07 (6:43 - 7:30)
Daily energy intake (kcal)	2011 (303)	1835 (262) *	1916 (383)
Protein (EN%)	15.45 (1.98)	15.06 (1.90)	15.44 (2.07)
Carbohydrates (EN%)	42.86 (39.26 – 46.15)	43.83 (41.33 – 46.20)	43.33 (40.08 – 45.61)
Fat (EN%)	39.75 (36.38 – 42.00)	39.39 (36.41 – 42.71)	39.39 (37.66 – 42.89)
Physical activity (MET)	1.59 (0.15)	1.56 (0.14)	1.57 (0.12)

Abbreviations: kcal, kilo calories; EN%, energy percent; MET, metabolic equivalent of task. For parameters with normal distribution mean (SD) are reported, for parameters with non-normal distribution median (25th IQR - 75th IQR) are reported. * $p<0.001$ in a paired T-test.

Table S3. Changes of the body weight, body composition, and blood lipids within eTRE and lTRE interventions.

**Characteristics**	**Before eTRE, mean (SD)**	**After eTRE, mean (SD)**	**Change eTRE after – before (95% CI)**	**P[a)]**	**Before lTRE, mean (SD)**	**After lTRE mean (SD)**	**Change lTRE After – before (95% CI)**	**P[a)]**	**Difference between lTRE vs. eTRE (95% CI)[a]**	**P[a)]**
BMI (kg/m^2)	30.4 (2.9)	30.0 (2.8)	-0.45 (-0.56 to -0.33)	**$5.4x10^{-9}$**	30.3 (2.8)	30.2 (2.9)	-0.12 (-0.21 to -0.03)	**0.01**	0.33 (0.21 to 0.44)	**$2.3x10^{-6}$**
Weight (kg)	82.3 (8.4)	81.3 (8.0)	-1.08 (-1.40 to -0.77)	**$9.1x10^{-8}$**	82.0 (8.1)	81.5 (8.1)	-0.44 (-0.74 to -0.13)	**0.01**	0.65 (0.27 to 1.03)	**0.002**
Fat mass (kg)	34.6 (5.5)	34.0 (5.8)	-0.61 (-1.01 to -0.22)	**0.002**	34.1 (5.6)	34.1 (5.5)	0.02 (-0.36 to 0.39)	0.92	0.65 (0.19 to 1.11)	**0.007**
Lean mass (kg)	47.9 (4.5)	47.4 (3.8)	-0.57 (-1.11 to -0.04)	**0.04**	47.8 (4.3)	47.5 (4.3)	-0.28 (-0.78 to 0.23)	0.27	0.25 (-0.31 to 0.81)	0.37
Waist circumference (cm)	99.1 (9.5)	98.8 (8.2)	-0.36 (-1.65 to 0.92)	0.57	98.9 (8.2)	99.3 (8.3)	0.48 (-0.97 to 1.92)	0.51	0.84 (-1.21 to 2.89)	0.41
Total cholesterol, mmol/L	5.54 (0.92)	5.42 (0.83)	-0.12 (-0.32 to 0.08)	0.24	5.73 (1.05)	5.62 (0.92)	-0.12 (-0.30 to 0.06)	0.19	0.001 (-0.28 to 0.28)	0.94
HDL cholesterol, mmol/L	1.56 (0.38)	1.46 (0.33)	-0.10 (-0.14 to -0.06)	**$6.0x10^{-5}$**	1.57 (0.37)	1.50 (0.38)	-0.07 (-0.11 to -0.03)	**0.003**	0.03 (-0.04 to 0.09)	0.45
LDL cholesterol, mmol/L	3.71 (1.12)	3.92 (1.12)	0.21 (-0.15 to 0.57)	0.24	3.87 (1.26)	4.09 (1.18)	0.22 (-0.10 to 0.55)	0.17	0.01 (-0.54 to 0.56)	0.96
Triglycerides, mmol/L	1.29 (0.53)	1.36 (0.58)	0.06 (-0.08 to 0.21)	0.27	1.36 (0.61)	1.35 (0.70)	-0.01 (-0.15 to 0.13)	0.56	-0.07 (-0.22 to 0.08)	0.34

Data are shown as mean (SD) or mean (95% CI).

[a)] Comparison by paired Student's t-test for parameters with normal distribution or Wilcoxon signed-rank test for parameters with non-normal distribution.

Table S4. All identified lipid classes and species.

A. Classes

cholesterol (Chol)
cholesterol esters (CE)
triacylglycerols (TAG)
diacylglycerols (DAG)
phosphatidylcholines (PCs
phosphatidylcholine ethers (PC O-)
phosphatidylethanolamines (PE)
phosphatidylethanolamine ethers (PE O-)
phosphatidylinositols (PI)
lysophosphatidylcholines (LPC)
lysophosphatidylcholine ethers (LPC O-)
lysophosphatidylethanolamines (LPE)
sphingomyelins (SM)
ceramides (CER)

B. Species

**feature**	**Shorthand Notation**	**SwissLipids Name**	**SwissLipids ID**
CE 14:0;0	SE 27:1/14:0	tetradecanoyl-cholesterol	SLM:000500261
CE 15:0;0	SE 27:1/15:0	pentadecanoyl-cholesterol	SLM:000500263
CE 16:0;0	SE 27:1/16:0	hexadecanoyl-cholesterol	SLM:000389778
CE 16:1;0	SE 27:1/16:1	Sterol ester (27:1/16:1)	SLM:000500345
CE 17:0;0	SE 27:1/17:0	heptadecanoyl-cholesterol	SLM:000500268
CE 17:1;0	SE 27:1/17:1	Sterol ester (27:1/17:1)	
CE 18:0;0	SE 27:1/18:0	octadecanoyl-cholesterol	SLM:000500276
CE 18:1;0	SE 27:1/18:1	Sterol ester (27:1/18:1)	SLM:000500351
CE 18:2;0	SE 27:1/18:2	Sterol ester (27:1/18:2)	SLM:000500350
CE 18:3;0	SE 27:1/18:3	Sterol ester (27:1/18:3)	SLM:000500349
CE 20:2;0	SE 27:1/20:2	Sterol ester (27:1/20:2)	SLM:000500357
CE 20:3;0	SE 27:1/20:3	Sterol ester (27:1/20:3)	SLM:000500356

CE 20:4;0	SE 27:1/20:4	Sterol ester (27:1/20:4)	SLM:000500355
CE 20:5;0	SE 27:1/20:5	Sterol ester (27:1/20:5)	SLM:000500354
CE 22:6;0	SE 27:1/22:6	Sterol ester (27:1/22:6)	SLM:000500361
Cer 40:0;2	Cer 40:0;O2	Ceramide (d40:0)	SLM:000391322
Cer 40:1;2	Cer 40:1;O2	Ceramide (d40:1)	SLM:000391319
Cer 40:2;2	Cer 40:2;O2	Ceramide (d40:2)	SLM:000391317
Cer 42:0;2	Cer 42:0;O2	Ceramide (d42:0)	SLM:000391349
Cer 42:1;2	Cer 42:1;O2	Ceramide (d42:1)	SLM:000391346
Cer 42:2;2	Cer 42:2;O2	Ceramide (d42:2)	SLM:000391345
Chol	ST 27:1;O	cholesterol	SLM:000000287
DAG 16:0;0_18:1;0	DG 16:0_18:1	Diacylglycerol (16:0_18:1)	SLM:000308862
DAG 16:0;0_18:2;0	DG 16:0_18:2	Diacylglycerol (16:0_18:2)	SLM:000308863
DAG 16:1;0_18:1;0	DG 16:1_18:1	Diacylglycerol (16:1_18:1)	SLM:000308894
DAG 18:1;0_18:1;0	DG 18:1_18:1	Diacylglycerol (18:1_18:1)	SLM:000309012
DAG 18:1;0_18:2;0	DG 18:1_18:2	Diacylglycerol (18:1_18:2)	SLM:000309013
DAG 18:1;0_18:3;0	DG 18:1_18:3	Diacylglycerol (18:1_18:3)	SLM:000309014
HexCer 40:1;2	HexCer 40:1;O2	Hexosyl ceramide (d40:1)	SLM:000390387
HexCer 42:1;2	HexCer 42:1;O2	Hexosyl ceramide (d42:1)	SLM:000390412
HexCer 42:2;2	HexCer 42:2;O2	Hexosyl ceramide (d42:2)	SLM:000390411
LPC 16:0;0	LPC 16:0	Phosphatidylcholine (16:0_0:0)	SLM:000063723
LPC 16:1;0	LPC 16:1	Phosphatidylcholine (16:1_0:0)	SLM:000063777
LPC 18:0;0	LPC 18:0	Phosphatidylcholine (18:0_0:0)	SLM:000063933
LPC 18:1;0	LPC 18:1	Phosphatidylcholine (18:1_0:0)	SLM:000063983
LPC 18:2;0	LPC 18:2	Phosphatidylcholine (18:2_0:0)	SLM:000064032
LPC 20:3;0	LPC 20:3	Phosphatidylcholine (20:3_0:0)	SLM:000064347
LPC 20:4;0	LPC 20:4	Phosphatidylcholine (20:4_0:0)	SLM:000064388
LPE 16:0;0	LPE 16:0	Phosphatidylethanolamine (16:0_0:0)	SLM:000067687
LPE 18:0;0	LPE 18:0	Phosphatidylethanolamine (18:0_0:0)	SLM:000067897
LPE 18:1;0	LPE 18:1	Phosphatidylethanolamine (18:1_0:0)	SLM:000067947

LPE 18:2;0	LPE 18:2	Phosphatidylethanolamine (18:2_0:0)	SLM:000067996
LPE 20:0;0	LPE 20:0	Phosphatidylethanolamine (20:0_0:0)	SLM:000068182
LPE 20:1;0	LPE 20:1	Phosphatidylethanolamine (20:1_0:0)	SLM:000068226
LPE 20:2;0	LPE 20:2	Phosphatidylethanolamine (20:2_0:0)	SLM:000068269
LPE 20:4;0	LPE 20:4	Phosphatidylethanolamine (20:4_0:0)	SLM:000068352
LPE 22:6;0	LPE 22:6	Phosphatidylethanolamine (22:6_0:0)	SLM:000068676
PC 14:0;0_16:0;0	PC 14:0_16:0	Phosphatidylcholine (14:0_16:0)	SLM:000063559
PC 14:0;0_18:0;0	PC 14:0_18:0	Phosphatidylcholine (14:0_18:0)	SLM:000063563
PC 14:0;0_18:1;0	PC 14:0_18:1	Phosphatidylcholine (14:0_18:1)	SLM:000063564
PC 14:0;0_18:2;0	PC 14:0_18:2	Phosphatidylcholine (14:0_18:2)	SLM:000063565
PC 14:0;0_20:3;0	PC 14:0_20:3	Phosphatidylcholine (14:0_20:3)	SLM:000063572
PC 14:0;0_20:4;0	PC 14:0_20:4	Phosphatidylcholine (14:0_20:4)	SLM:000063573
PC 15:0;0_16:0;0	PC 15:0_16:0	Phosphatidylcholine (15:0_16:0)	SLM:000063670
PC 15:0;0_17:0;0	PC 15:0_17:0	Phosphatidylcholine (15:0_17:0)	SLM:000063673
PC 15:0;0_18:1;0	PC 15:0_18:1	Phosphatidylcholine (15:0_18:1)	SLM:000063675
PC 15:0;0_18:2;0	PC 15:0_18:2	Phosphatidylcholine (15:0_18:2)	SLM:000063676
PC 15:0;0_20:3;0	PC 15:0_20:3	Phosphatidylcholine (15:0_20:3)	SLM:000063683
PC 15:0;0_20:4;0	PC 15:0_20:4	Phosphatidylcholine (15:0_20:4)	SLM:000063684
PC 16:0;0_16:0;0	PC 16:0_16:0	Phosphatidylcholine (16:0/16:0)	SLM:000088143
PC 16:0;0_16:1;0	PC 16:0_16:1	Phosphatidylcholine (16:0_16:1)	SLM:000063725
PC 16:0;0_17:1;0	PC 16:0_17:1	Phosphatidylcholine (16:0_17:1)	
PC 16:0;0_18:0;0	PC 16:0_18:0	Phosphatidylcholine (16:0_18:0)	SLM:000063728
PC 16:0;0_18:1;0	PC 16:0_18:1	Phosphatidylcholine (16:0_18:1)	SLM:000063729
PC 16:0;0_18:2;0	PC 16:0_18:2	Phosphatidylcholine (16:0_18:2)	SLM:000063730
PC 16:0;0_18:3;0	PC 16:0_18:3	Phosphatidylcholine (16:0_18:3)	SLM:000063731
PC 16:0;0_19:1;0	PC 16:0_19:1	Phosphatidylcholine (16:0_19:1)	
PC 16:0;0_20:1;0	PC 16:0_20:1	Phosphatidylcholine (16:0_20:1)	SLM:000063735
PC 16:0;0_20:2;0	PC 16:0_20:2	Phosphatidylcholine (16:0_20:2)	SLM:000063736

PC 16:0;0_20:3;0	PC 16:0_20:3	Phosphatidylcholine (16:0_20:3)	SLM:000063737
PC 16:0;0_20:4;0	PC 16:0_20:4	Phosphatidylcholine (16:0_20:4)	SLM:000063738
PC 16:0;0_20:5;0	PC 16:0_20:5	Phosphatidylcholine (16:0_20:5)	SLM:000063739
PC 16:0;0_22:4;0	PC 16:0_22:4	Phosphatidylcholine (16:0_22:4)	SLM:000063745
PC 16:0;0_22:5;0	PC 16:0_22:5	Phosphatidylcholine (16:0_22:5)	SLM:000063746
PC 16:0;0_22:6;0	PC 16:0_22:6	Phosphatidylcholine (16:0_22:6)	SLM:000063747
PC 16:1;0_16:1;0	PC 16:1_16:1	Phosphatidylcholine (16:1/16:1)	SLM:000088209
PC 16:1;0_17:0;0	PC 16:1_17:0	Phosphatidylcholine (16:1_17:0)	SLM:000063780
PC 16:1;0_18:0;0	PC 16:1_18:0	Phosphatidylcholine (16:1_18:0)	SLM:000063781
PC 16:1;0_18:1;0	PC 16:1_18:1	Phosphatidylcholine (16:1_18:1)	SLM:000063782
PC 16:1;0_18:2;0	PC 16:1_18:2	Phosphatidylcholine (16:1_18:2)	SLM:000063783
PC 16:1;0_20:3;0	PC 16:1_20:3	Phosphatidylcholine (16:1_20:3)	SLM:000063790
PC 16:1;0_20:4;0	PC 16:1_20:4	Phosphatidylcholine (16:1_20:4)	SLM:000063791
PC 17:0;0_18:1;0	PC 17:0_18:1	Phosphatidylcholine (17:0_18:1)	SLM:000063885
PC 17:0;0_18:2;0	PC 17:0_18:2	Phosphatidylcholine (17:0_18:2)	SLM:000063886
PC 17:0;0_18:3;0	PC 17:0_18:3	Phosphatidylcholine (17:0_18:3)	SLM:000063887
PC 17:0;0_20:3;0	PC 17:0_20:3	Phosphatidylcholine (17:0_20:3)	SLM:000063893
PC 17:0;0_20:4;0	PC 17:0_20:4	Phosphatidylcholine (17:0_20:4)	SLM:000063894
PC 17:0;0_20:5;0	PC 17:0_20:5	Phosphatidylcholine (17:0_20:5)	SLM:000063895
PC 17:0;0_20:6;0	PC 17:0_20:6	Phosphatidylcholine (17:0_20:6)	
PC 17:0;0_22:4;0	PC 17:0_22:4	Phosphatidylcholine (17:0_22:4)	SLM:000063901
PC 17:0;0_22:5;0	PC 17:0_22:5	Phosphatidylcholine (17:0_22:5)	SLM:000063902
PC 17:0;0_22:6;0	PC 17:0_22:6	Phosphatidylcholine (17:0_22:6)	SLM:000063903
PC 17:1;0_18:0;0	PC 17:1_18:0	Phosphatidylcholine (17:1_18:0)	
PC 17:1;0_18:1;0	PC 17:1_18:1	Phosphatidylcholine (17:1_18:1)	
PC 17:1;0_18:2;0	PC 17:1_18:2	Phosphatidylcholine (17:1_18:2)	
PC 18:0;0_18:1;0	PC 18:0_18:1	Phosphatidylcholine (18:0_18:1)	SLM:000063935
PC 18:0;0_18:2;0	PC 18:0_18:2	Phosphatidylcholine (18:0_18:2)	SLM:000063936
PC 18:0;0_18:3;0	PC 18:0_18:3	Phosphatidylcholine (18:0_18:3)	SLM:000063937
PC 18:0;0_20:2;0	PC 18:0_20:2	Phosphatidylcholine (18:0_20:2)	SLM:000063942
PC 18:0;0_20:3;0	PC 18:0_20:3	Phosphatidylcholine (18:0_20:3)	SLM:000063943

PC 18:0;0_20:4;0	PC 18:0_20:4	Phosphatidylcholine (18:0_20:4)	SLM:000063944
PC 18:0;0_20:5;0	PC 18:0_20:5	Phosphatidylcholine (18:0_20:5)	SLM:000063945
PC 18:0;0_22:4;0	PC 18:0_22:4	Phosphatidylcholine (18:0_22:4)	SLM:000063951
PC 18:0;0_22:5;0	PC 18:0_22:5	Phosphatidylcholine (18:0_22:5)	SLM:000063952
PC 18:0;0_22:6;0	PC 18:0_22:6	Phosphatidylcholine (18:0_22:6)	SLM:000063953
PC 18:1;0_18:1;0	PC 18:1_18:1	Phosphatidylcholine (18:1/18:1)	SLM:000088473
PC 18:1;0_18:2;0	PC 18:1_18:2	Phosphatidylcholine (18:1_18:2)	SLM:000063985
PC 18:1;0_18:3;0	PC 18:1_18:3	Phosphatidylcholine (18:1_18:3)	SLM:000063986
PC 18:1;0_20:1;0	PC 18:1_20:1	Phosphatidylcholine (18:1_20:1)	SLM:000063990
PC 18:1;0_20:2;0	PC 18:1_20:2	Phosphatidylcholine (18:1_20:2)	SLM:000063991
PC 18:1;0_20:3;0	PC 18:1_20:3	Phosphatidylcholine (18:1_20:3)	SLM:000063992
PC 18:1;0_20:4;0	PC 18:1_20:4	Phosphatidylcholine (18:1_20:4)	SLM:000063993
PC 18:1;0_20:5;0	PC 18:1_20:5	Phosphatidylcholine (18:1_20:5)	SLM:000063994
PC 18:1;0_22:5;0	PC 18:1_22:5	Phosphatidylcholine (18:1_22:5)	SLM:000064001
PC 18:1;0_22:6;0	PC 18:1_22:6	Phosphatidylcholine (18:1_22:6)	SLM:000064002
PC 18:2;0_18:2;0	PC 18:2_18:2	Phosphatidylcholine (18:2/18:2)	SLM:000088539
PC 18:2;0_18:3;0	PC 18:2_18:3	Phosphatidylcholine (18:2_18:3)	SLM:000064034
PC 18:2;0_19:0;0	PC 18:2_19:0	Phosphatidylcholine (18:2_19:0)	SLM:000064036
PC 18:2;0_20:0;0	PC 18:2_20:0	Phosphatidylcholine (18:2_20:0)	SLM:000064037
PC 18:2;0_20:1;0	PC 18:2_20:1	Phosphatidylcholine (18:2_20:1)	SLM:000064038
PC 18:2;0_20:2;0	PC 18:2_20:2	Phosphatidylcholine (18:2_20:2)	SLM:000064039
PC 18:2;0_20:3;0	PC 18:2_20:3	Phosphatidylcholine (18:2_20:3)	SLM:000064040
PC 18:2;0_20:4;0	PC 18:2_20:4	Phosphatidylcholine (18:2_20:4)	SLM:000064041
PC 20:0;0_20:4;0	PC 20:0_20:4	Phosphatidylcholine (20:0_20:4)	SLM:000064223
PC 20:1;0_20:4;0	PC 20:1_20:4	Phosphatidylcholine (20:1_20:4)	SLM:000064266
PC 20:3;0_20:4;0	PC 20:3_20:4	Phosphatidylcholine (20:3_20:4)	SLM:000064349
PC O-16:0;0/16:0;0	PC O-16:0/16:0	Phosphatidylcholine (O-16:0/16:0)	SLM:000092108
PC O-16:0;0/16:1;0	PC O-16:0/16:1	Phosphatidylcholine (O-16:0/16:1)	SLM:000092109
PC O-16:0;0/18:0;0	PC O-16:0/18:0	Phosphatidylcholine (O-16:0/18:0)	SLM:000092112
PC O-16:0;0/18:1;0	PC O-16:0/18:1	Phosphatidylcholine (O-16:0/18:1)	SLM:000092113
PC O-16:0;0/18:2;0	PC O-16:0/18:2	Phosphatidylcholine (O-16:0/18:2)	SLM:000092114

PC O-16:0;0/18:3;0	PC O-16:0/18:3	Phosphatidylcholine (O-16:0/18:3)	SLM:000092115
PC O-16:0;0/20:3;0	PC O-16:0/20:3	Phosphatidylcholine (O-16:0/20:3)	SLM:000092121
PC O-16:0;0/20:4;0	PC O-16:0/20:4	Phosphatidylcholine (O-16:0/20:4)	SLM:000092122
PC O-16:0;0/20:5;0	PC O-16:0/20:5	Phosphatidylcholine (O-16:0/20:5)	SLM:000092123
PC O-16:0;0/22:4;0	PC O-16:0/22:4	Phosphatidylcholine (O-16:0/22:4)	SLM:000092129
PC O-16:0;0/22:5;0	PC O-16:0/22:5	Phosphatidylcholine (O-16:0/22:5)	SLM:000092130
PC O-16:0;0/22:6;0	PC O-16:0/22:6	Phosphatidylcholine (O-16:0/22:6)	SLM:000092131
PC O-16:1;0/16:0;0	PC O-16:1/16:0	Phosphatidylcholine (O-16:1_16:0)	SLM:000065984
PC O-16:1;0/16:1;0	PC O-16:1/16:1	Phosphatidylcholine (O-16:1_16:1)	SLM:000065985
PC O-16:1;0/18:0;0	PC O-16:1/18:0	Phosphatidylcholine (O-16:1_18:0)	SLM:000065988
PC O-16:1;0/18:1;0	PC O-16:1/18:1	Phosphatidylcholine (O-16:1_18:1)	SLM:000065989
PC O-16:1;0/18:2;0	PC O-16:1/18:2	Phosphatidylcholine (O-16:1_18:2)	SLM:000065990
PC O-16:1;0/20:3;0	PC O-16:1/20:3	Phosphatidylcholine (O-16:1_20:3)	SLM:000065997
PC O-16:1;0/20:4;0	PC O-16:1/20:4	Phosphatidylcholine (O-16:1_20:4)	SLM:000065998
PC O-16:1;0/22:4;0	PC O-16:1/22:4	Phosphatidylcholine (O-16:1_22:4)	SLM:000066005
PC O-16:1;0/22:5;0	PC O-16:1/22:5	Phosphatidylcholine (O-16:1_22:5)	SLM:000066006
PC O-16:2;0/16:0;0	PC O-16:2/16:0	Phosphatidylcholine (O-16:2_16:0)	
PC O-16:2;0/18:0;0	PC O-16:2/18:0	Phosphatidylcholine (O-16:2_18:0)	
PC O-16:2;0/18:1;0	PC O-16:2/18:1	Phosphatidylcholine (O-16:2_18:1)	
PC O-17:0;0/15:0;0	PC O-17:0/15:0	Phosphatidylcholine (O-17:0/15:0)	SLM:000092172
PC O-17:0;0/17:0;0	PC O-17:0/17:0	Phosphatidylcholine (O-17:0/17:0)	SLM:000092176
PC O-17:0;0/17:1;0	PC O-17:0/17:1	Phosphatidylcholine (O-17:0/17:1)	
PC O-17:1;0/17:0;0	PC O-17:1/17:0	Phosphatidylcholine (O-17:1_17:0)	SLM:000066117
PC O-17:2;0/17:0;0	PC O-17:2/17:0	Phosphatidylcholine (O-17:2_17:0)	
PC O-18:0;0/14:0;0	PC O-18:0/14:0	Phosphatidylcholine (O-18:0/14:0)	SLM:000092235
PC O-18:0;0/16:0;0	PC O-18:0/16:0	Phosphatidylcholine (O-18:0/16:0)	SLM:000092238
PC O-18:0;0/16:1;0	PC O-18:0/16:1	Phosphatidylcholine (O-18:0/16:1)	SLM:000092239
PC O-18:0;0/18:2;0	PC O-18:0/18:2	Phosphatidylcholine (O-18:0/18:2)	SLM:000092244
PC O-18:0;0/18:3;0	PC O-18:0/18:3	Phosphatidylcholine (O-18:0/18:3)	SLM:000092245
PC O-18:0;0/20:4;0	PC O-18:0/20:4	Phosphatidylcholine (O-18:0/20:4)	SLM:000092252
PC O-18:0;0/20:5;0	PC O-18:0/20:5	Phosphatidylcholine (O-18:0/20:5)	SLM:000092253

PC O-18:0;0/20:6;0	PC O-18:0/20:6	Phosphatidylcholine (O-18:0/20:6)	
PC O-18:0;0/22:5;0	PC O-18:0/22:5	Phosphatidylcholine (O-18:0/22:5)	SLM:000092260
PC O-18:0;0/22:6;0	PC O-18:0/22:6	Phosphatidylcholine (O-18:0/22:6)	SLM:000092261
PC O-18:1;0/16:0;0	PC O-18:1/16:0	Phosphatidylcholine (O-18:1_16:0)	SLM:000066244
PC O-18:1;0/16:1;0	PC O-18:1/16:1	Phosphatidylcholine (O-18:1_16:1)	SLM:000066245
PC O-18:1;0/18:1;0	PC O-18:1/18:1	Phosphatidylcholine (O-18:1_18:1)	SLM:000066249
PC O-18:1;0/18:2;0	PC O-18:1/18:2	Phosphatidylcholine (O-18:1_18:2)	SLM:000066250
PC O-18:1;0/20:3;0	PC O-18:1/20:3	Phosphatidylcholine (O-18:1_20:3)	SLM:000066257
PC O-18:1;0/20:4;0	PC O-18:1/20:4	Phosphatidylcholine (O-18:1_20:4)	SLM:000066258
PC O-18:1;0/22:4;0	PC O-18:1/22:4	Phosphatidylcholine (O-18:1_22:4)	SLM:000066265
PC O-18:1;0/22:5;0	PC O-18:1/22:5	Phosphatidylcholine (O-18:1_22:5)	SLM:000066266
PC O-18:2;0/16:0;0	PC O-18:2/16:0	Phosphatidylcholine (O-18:2_16:0)	SLM:000066309
PC O-18:2;0/18:0;0	PC O-18:2/18:0	Phosphatidylcholine (O-18:2_18:0)	SLM:000066313
PC O-18:2;0/18:1;0	PC O-18:2/18:1	Phosphatidylcholine (O-18:2_18:1)	SLM:000066314
PC O-18:2;0/18:2;0	PC O-18:2/18:2	Phosphatidylcholine (O-18:2_18:2)	SLM:000066315
PC O-18:2;0/20:3;0	PC O-18:2/20:3	Phosphatidylcholine (O-18:2_20:3)	SLM:000066322
PC O-18:2;0/20:4;0	PC O-18:2/20:4	Phosphatidylcholine (O-18:2_20:4)	SLM:000066323
PE 16:0;0_18:1;0	PE 16:0_18:1	Phosphatidylethanolamine (16:0_18:1)	SLM:000067693
PE 16:0;0_18:2;0	PE 16:0_18:2	Phosphatidylethanolamine (16:0_18:2)	SLM:000067694
PE 16:0;0_20:3;0	PE 16:0_20:3	Phosphatidylethanolamine (16:0_20:3)	SLM:000067701
PE 16:0;0_20:4;0	PE 16:0_20:4	Phosphatidylethanolamine (16:0_20:4)	SLM:000067702
PE 16:0;0_22:5;0	PE 16:0_22:5	Phosphatidylethanolamine (16:0_22:5)	SLM:000067710
PE 16:0;0_22:6;0	PE 16:0_22:6	Phosphatidylethanolamine (16:0_22:6)	SLM:000067711
PE 16:1;0_18:0;0	PE 16:1_18:0	Phosphatidylethanolamine (16:1_18:0)	SLM:000067745
PE 18:0;0_18:1;0	PE 18:0_18:1	Phosphatidylethanolamine (18:0_18:1)	SLM:000067899
PE 18:0;0_18:2;0	PE 18:0_18:2	Phosphatidylethanolamine (18:0_18:2)	SLM:000067900

PE 18:0;0_18:3;0	PE 18:0_18:3	Phosphatidylethanolamine (18:0_18:3)	SLM:000067901
PE 18:0;0_20:2;0	PE 18:0_20:2	Phosphatidylethanolamine (18:0_20:2)	SLM:000067906
PE 18:0;0_20:3;0	PE 18:0_20:3	Phosphatidylethanolamine (18:0_20:3)	SLM:000067907
PE 18:0;0_20:4;0	PE 18:0_20:4	Phosphatidylethanolamine (18:0_20:4)	SLM:000067908
PE 18:0;0_20:5;0	PE 18:0_20:5	Phosphatidylethanolamine (18:0_20:5)	SLM:000067909
PE 18:0;0_22:4;0	PE 18:0_22:4	Phosphatidylethanolamine (18:0_22:4)	SLM:000067915
PE 18:0;0_22:5;0	PE 18:0_22:5	Phosphatidylethanolamine (18:0_22:5)	SLM:000067916
PE 18:0;0_22:6;0	PE 18:0_22:6	Phosphatidylethanolamine (18:0_22:6)	SLM:000067917
PE 18:1;0_18:1;0	PE 18:1_18:1	Phosphatidylethanolamine (18:1/18:1)	SLM:000094777
PE 18:1;0_18:2;0	PE 18:1_18:2	Phosphatidylethanolamine (18:1_18:2)	SLM:000067949
PE 18:1;0_20:3;0	PE 18:1_20:3	Phosphatidylethanolamine (18:1_20:3)	SLM:000067956
PE 18:1;0_20:4;0	PE 18:1_20:4	Phosphatidylethanolamine (18:1_20:4)	SLM:000067957
PE 18:2;0_18:2;0	PE 18:2_18:2	Phosphatidylethanolamine (18:2/18:2)	SLM:000094843
PE O-16:0;0/18:2;0	PE O-16:0/18:2	Phosphatidylethanolamine (O-16:0/18:2)	SLM:000098418
PE O-16:0;0/20:4;0	PE O-16:0/20:4	Phosphatidylethanolamine (O-16:0/20:4)	SLM:000098426
PE O-16:0;0/22:5;0	PE O-16:0/22:5	Phosphatidylethanolamine (O-16:0/22:5)	SLM:000098434
PE O-16:1;0/18:1;0	PE O-16:1/18:1	Phosphatidylethanolamine (O-16:1_18:1)	SLM:000069953
PE O-16:1;0/18:2;0	PE O-16:1/18:2	Phosphatidylethanolamine (O-16:1_18:2)	SLM:000069954
PE O-16:1;0/20:3;0	PE O-16:1/20:3	Phosphatidylethanolamine (O-16:1_20:3)	SLM:000069961
PE O-16:1;0/20:4;0	PE O-16:1/20:4	Phosphatidylethanolamine	SLM:000069962

		(O-16:1_20:4)	
PE O-16:1;0/22:4;0	PE O-16:1/22:4	Phosphatidylethanolamine (O-16:1_22:4)	SLM:000069969
PE O-16:1;0/22:5;0	PE O-16:1/22:5	Phosphatidylethanolamine (O-16:1_22:5)	SLM:000069970
PE O-16:1;0/22:6;0	PE O-16:1/22:6	Phosphatidylethanolamine (O-16:1_22:6)	SLM:000069971
PE O-16:2;0/18:0;0	PE O-16:2/18:0	Phosphatidylethanolamine (O-16:2_18:0)	
PE O-18:0;0/18:2;0	PE O-18:0/18:2	Phosphatidylethanolamine (O-18:0/18:2)	SLM:000098548
PE O-18:0;0/20:4;0	PE O-18:0/20:4	Phosphatidylethanolamine (O-18:0/20:4)	SLM:000098556
PE O-18:1;0/18:1;0	PE O-18:1/18:1	Phosphatidylethanolamine (O-18:1_18:1)	SLM:000070213
PE O-18:1;0/18:2;0	PE O-18:1/18:2	Phosphatidylethanolamine (O-18:1_18:2)	SLM:000070214
PE O-18:1;0/20:3;0	PE O-18:1/20:3	Phosphatidylethanolamine (O-18:1_20:3)	SLM:000070221
PE O-18:1;0/20:4;0	PE O-18:1/20:4	Phosphatidylethanolamine (O-18:1_20:4)	SLM:000070222
PE O-18:1;0/20:5;0	PE O-18:1/20:5	Phosphatidylethanolamine (O-18:1_20:5)	SLM:000070223
PE O-18:1;0/22:4;0	PE O-18:1/22:4	Phosphatidylethanolamine (O-18:1_22:4)	SLM:000070229
PE O-18:1;0/22:6;0	PE O-18:1/22:6	Phosphatidylethanolamine (O-18:1_22:6)	SLM:000070231
PE O-18:2;0/16:0;0	PE O-18:2/16:0	Phosphatidylethanolamine (O-18:2_16:0)	SLM:000070273
PE O-18:2;0/18:0;0	PE O-18:2/18:0	Phosphatidylethanolamine (O-18:2_18:0)	SLM:000070277
PE O-18:2;0/18:1;0	PE O-18:2/18:1	Phosphatidylethanolamine (O-18:2_18:1)	SLM:000070278
PE O-18:2;0/18:2;0	PE O-18:2/18:2	Phosphatidylethanolamine (O-18:2_18:2)	SLM:000070279
PE O-18:2;0/20:3;0	PE O-18:2/20:3	Phosphatidylethanolamine (O-18:2_20:3)	SLM:000070286
PE O-18:2;0/20:4;0	PE O-18:2/20:4	Phosphatidylethanolamine (O-18:2_20:4)	SLM:000070287

PE O-18:2;0/22:5;0	PE O-18:2/22:5	Phosphatidylethanolamine (O-18:2_22:5)	SLM:000070295
PI 16:0;0_18:1;0	PI 16:0_18:1	Phosphatidylinositol (16:0_18:1)	SLM:000073801
PI 16:0;0_18:2;0	PI 16:0_18:2	Phosphatidylinositol (16:0_18:2)	SLM:000073802
PI 16:0;0_20:3;0	PI 16:0_20:3	Phosphatidylinositol (16:0_20:3)	SLM:000073809
PI 16:0;0_20:4;0	PI 16:0_20:4	Phosphatidylinositol (16:0_20:4)	SLM:000073810
PI 16:1;0_18:0;0	PI 16:1_18:0	Phosphatidylinositol (16:1_18:0)	SLM:000073853
PI 18:0;0_18:1;0	PI 18:0_18:1	Phosphatidylinositol (18:0_18:1)	SLM:000074007
PI 18:0;0_18:2;0	PI 18:0_18:2	Phosphatidylinositol (18:0_18:2)	SLM:000074008
PI 18:0;0_20:3;0	PI 18:0_20:3	Phosphatidylinositol (18:0_20:3)	SLM:000074015
PI 18:0;0_20:4;0	PI 18:0_20:4	Phosphatidylinositol (18:0_20:4)	SLM:000074016
PI 18:0;0_22:5;0	PI 18:0_22:5	Phosphatidylinositol (18:0_22:5)	SLM:000074024
PI 18:1;0_18:1;0	PI 18:1_18:1	Phosphatidylinositol (18:1/18:1)	SLM:000105305
PI 18:1;0_18:2;0	PI 18:1_18:2	Phosphatidylinositol (18:1_18:2)	SLM:000074057
PI 18:1;0_20:3;0	PI 18:1_20:3	Phosphatidylinositol (18:1_20:3)	SLM:000074064
PI 18:1;0_20:4;0	PI 18:1_20:4	Phosphatidylinositol (18:1_20:4)	SLM:000074065
PI 18:2;0_18:2;0	PI 18:2_18:2	Phosphatidylinositol (18:2/18:2)	SLM:000105371
SM 32:1;2	SM 32:1;O2	Sphingomyelin (d32:1)	SLM:000390695
SM 32:2;2	SM 32:2;O2	Sphingomyelin (d32:2)	SLM:000390694
SM 34:0;2	SM 34:0;O2	Sphingomyelin (d34:0)	SLM:000390716
SM 34:1;2	SM 34:1;O2	Sphingomyelin (d34:1)	SLM:000390714
SM 34:1;3	SM 34:1;O3	Sphingomyelin (t34:1)	SLM:000390728
SM 34:2;2	SM 34:2;O2	Sphingomyelin (d34:2)	SLM:000390712
SM 36:1;2	SM 36:1;O2	Sphingomyelin (d36:1)	SLM:000390739
SM 36:2;2	SM 36:2;O2	Sphingomyelin (d36:2)	SLM:000390737
SM 38:1;2	SM 38:1;O2	Sphingomyelin (d38:1)	SLM:000390767
SM 38:2;2	SM 38:2;O2	Sphingomyelin (d38:2)	SLM:000390765
SM 40:1;2	SM 40:1;O2	Sphingomyelin (d40:1)	SLM:000390797
SM 40:2;2	SM 40:2;O2	Sphingomyelin (d40:2)	SLM:000390795
SM 42:1;2	SM 42:1;O2	Sphingomyelin (d42:1)	SLM:000390824
SM 42:2;2	SM 42:2;O2	Sphingomyelin (d42:2)	SLM:000390823
TAG 44:1;0	TG 44:1	Triacylglycerol (44:1)	SLM:000308232

TAG 46:0;0	TG 46:0	Triacylglycerol (46:0)	SLM:000308243
TAG 46:1;0	TG 46:1	Triacylglycerol (46:1)	SLM:000308244
TAG 46:2;0	TG 46:2	Triacylglycerol (46:2)	SLM:000308245
TAG 48:0;0	TG 48:0	Triacylglycerol (48:0)	SLM:000308257
TAG 48:1;0	TG 48:1	Triacylglycerol (48:1)	SLM:000308258
TAG 48:2;0	TG 48:2	Triacylglycerol (48:2)	SLM:000308259
TAG 48:3;0	TG 48:3	Triacylglycerol (48:3)	SLM:000308260
TAG 49:1;0	TG 49:1	Triacylglycerol (49:1)	SLM:000308267
TAG 49:2;0	TG 49:2	Triacylglycerol (49:2)	SLM:000308268
TAG 49:3;0	TG 49:3	Triacylglycerol (49:3)	SLM:000308269
TAG 50:1;0	TG 50:1	Triacylglycerol (50:1)	SLM:000308276
TAG 50:2;0	TG 50:2	Triacylglycerol (50:2)	SLM:000308277
TAG 50:3;0	TG 50:3	Triacylglycerol (50:3)	SLM:000308278
TAG 50:4;0	TG 50:4	Triacylglycerol (50:4)	SLM:000308279
TAG 50:5;0	TG 50:5	Triacylglycerol (50:5)	SLM:000308280
TAG 51:1;0	TG 51:1	Triacylglycerol (51:1)	SLM:000308286
TAG 51:2;0	TG 51:2	Triacylglycerol (51:2)	SLM:000308287
TAG 51:3;0	TG 51:3	Triacylglycerol (51:3)	SLM:000308288
TAG 51:4;0	TG 51:4	Triacylglycerol (51:4)	SLM:000308289
TAG 52:2;0	TG 52:2	Triacylglycerol (52:2)	SLM:000308298
TAG 52:3;0	TG 52:3	Triacylglycerol (52:3)	SLM:000308299
TAG 52:4;0	TG 52:4	Triacylglycerol (52:4)	SLM:000308300
TAG 52:5;0	TG 52:5	Triacylglycerol (52:5)	SLM:000308301
TAG 52:6;0	TG 52:6	Triacylglycerol (52:6)	SLM:000308302
TAG 53:2;0	TG 53:2	Triacylglycerol (53:2)	SLM:000308309
TAG 53:3;0	TG 53:3	Triacylglycerol (53:3)	SLM:000308310
TAG 53:4;0	TG 53:4	Triacylglycerol (53:4)	SLM:000308311
TAG 53:5;0	TG 53:5	Triacylglycerol (53:5)	SLM:000308312
TAG 54:3;0	TG 54:3	Triacylglycerol (54:3)	SLM:000308323
TAG 54:4;0	TG 54:4	Triacylglycerol (54:4)	SLM:000308324
TAG 54:5;0	TG 54:5	Triacylglycerol (54:5)	SLM:000308325

TAG 54:6;0	TG 54:6	Triacylglycerol (54:6)	SLM:000308326
TAG 54:7;0	TG 54:7	Triacylglycerol (54:7)	SLM:000308327
TAG 56:3;0	TG 56:3	Triacylglycerol (56:3)	SLM:000308349
TAG 56:5;0	TG 56:5	Triacylglycerol (56:5)	SLM:000308351
TAG 56:6;0	TG 56:6	Triacylglycerol (56:6)	SLM:000308352
TAG 56:7;0	TG 56:7	Triacylglycerol (56:7)	SLM:000308353
TAG 56:8;0	TG 56:8	Triacylglycerol (56:8)	SLM:000308354
TAG 56:9;0	TG 56:9	Triacylglycerol (56:9)	SLM:000308355
TAG 58:7;0	TG 58:7	Triacylglycerol (58:7)	SLM:000308381
TAG 58:8;0	TG 58:8	Triacylglycerol (58:8)	SLM:000308382
TAG 58:9;0	TG 58:9	Triacylglycerol (58:9)	SLM:000308383

Table S5. Lipid species showing alterations within or between eTRE and lTRE interventions.

Lipid species	Before eTRE	After eTRE	Change eTRE after – before (95% CI)	P[a)]	P BH[b)]	Before lTRE	After lTRE	Change lTRE After – before (95% CI)	P[a)]	P BH[b)]	Difference between lTRE vs. eTRE (95% CI)[a]	P[a)]	P BH[b)]
**PC 18:0;0_20:3;0**	35.6	27.5	-8.1 (4.6 to 11)	**0.000002**	**0.0006**	35.9	32.9	-3.1 (0.86 to 6)	**0.015**	0.85	4.8 (-9.1 to -0.49)	**0.028**	0.24
**LPE 20:2;0**	0.53	0.39	-0.13 (0.069 to 0.17)	**0.0000057**	**0.00076**	0.47	0.5	0.034 (-0.077 to 0.043)	0.73	1	0.2 (-0.29 to -0.066)	**0.001**	0.17
**PC 18:2;0_20:3;0**	4	3.1	-0.96 (0.56 to 1.3)	**0.0000076**	**0.00076**	3.9	3.6	-0.3 (-0.14 to 0.72)	0.19	1	0.82 (-1.4 to -0.19)	**0.0065**	0.17
**PC 18:0;0_18:2;0**	234.9	191.4	-43 (23 to 60)	**0.000024**	**0.0014**	234	225.9	-8.1 (-8 to 26)	0.43	1	35 (-65 to 1.1)	0.067	0.33
**PE 18:0;0_20:3;0**	2.5	2.1	-0.41 (0.26 to 0.54)	**0.000024**	**0.0014**	2.4	2.3	-0.16 (-0.11 to 0.46)	0.3	1	0.24 (-0.56 to 0.11)	0.16	0.38
**LPE 18:1;0**	0.72	0.52	-0.2 (0.068 to 0.26)	**0.000028**	**0.0014**	0.67	0.7	0.032 (-0.13 to 0.078)	0.67	1	0.25 (-0.38 to -0.093)	**0.0022**	0.17
**PE O-18:1;0/20:5;0**	0.72	0.53	-0.19 (0.14 to 0.32)	**0.000044**	**0.0019**	0.71	0.74	0.038 (-0.18 to 0.11)	0.79	1	0.34 (-0.55 to -0.099)	**0.0074**	0.17
**PC 16:0;0_18:3;0**	11.2	7.3	-4 (1.1 to 3.3)	**0.000063**	**0.0023**	9.5	9.9	0.39 (-0.95 to 1.5)	0.54	1	4.3 (-4.4 to -0.019)	0.05	0.29
**PE 18:0;0_18:3;0**	0.3	0.22	-0.08 (0.036 to 0.1)	**0.000068**	**0.0023**	0.29	0.28	-0.016 (-0.005 to 0.056)	0.086	1	0.085 (-0.096 to 0.011)	0.17	0.39
**PC 18:0;0_22:5;0**	7.2	5.8	-1.4 (0.69 to 2)	**0.000089**	**0.0024**	7.4	6.8	-0.57 (0.12 to 1.2)	**0.016**	0.85	0.83 (-1.7 to 0.082)	0.088	0.33

**CE 18:3;0**	121.4	92.3	-29 (12 to 40)	**0.000089**	**0.0024**	122	117.2	-4.8 (-8.6 to 17)	0.52	1	24 (-50 to 3.9)	0.12	0.35
**PC 18:0;0_18:1;0**	39	30.8	-8.2 (3.3 to 12)	**0.00011**	**0.0028**	39.4	38.3	-1.2 (-2.1 to 5.1)	0.38	1	7.1 (-11 to -0.45)	**0.04**	0.25
**PC 18:0;0_20:2;0**	2.6	2.1	-0.49 (0.29 to 0.81)	**0.00012**	**0.0028**	2.8	2.4	-0.33 (0.053 to 0.51)	**0.02**	0.85	0.28 (-0.74 to 0.089)	0.12	0.35
**PC 18:0;0_18:3;0**	4.5	2.8	-1.7 (0.64 to 2.5)	**0.00013**	**0.0028**	3.8	3.8	0.039 (-0.42 to 0.76)	0.51	1	1.4 (-2.3 to 0.022)	0.076	0.33
**PC 16:0;0_18:0;0**	27	21.5	-5.5 (2 to 6)	**0.00017**	**0.0032**	25	24.1	-0.89 (-1.1 to 2.5)	0.49	1	4.6 (-6.5 to -0.71)	**0.023**	0.23
**PE O-18:1;0/18:1;0**	0.63	0.5	-0.13 (0.055 to 0.23)	**0.00017**	**0.0032**	0.64	0.59	-0.046 (-0.028 to 0.15)	0.18	1	0.11 (-0.22 to 0.022)	0.15	0.37
**SM 42:1;2**	18.1	16.2	-1.9 (0.82 to 3)	**0.00023**	**0.0037**	18	18.3	0.36 (-1.7 to 1.4)	0.92	1	2.3 (-4 to 0.31)	0.084	0.33
**PC 18:1;0_20:2;0**	1.5	1.2	-0.28 (0.15 to 0.42)	**0.00023**	**0.0037**	1.5	1.4	-0.12 (-0.093 to 0.23)	0.42	1	0.2 (-0.48 to 0.075)	0.11	0.35
**PE O-18:1;0/18:2;0**	2.6	2.0	-0.56 (0.28 to 0.82)	**0.00023**	**0.0037**	2.5	2.3	-0.2 (-0.073 to 0.45)	0.18	1	0.38 (-0.88 to 0.097)	0.12	0.35
**PE 18:0;0_20:2;0**	3.5	2.9	-0.66 (0.33 to 1.1)	**0.00026**	**0.0038**	3.3	3.2	-0.063 (-0.35 to 0.33)	0.94	1	0.72 (-1.5 to -0.039)	**0.035**	0.24
**PC 18:2;0_18:2;0**	28.9	21.7	-7.2 (3.5 to 10)	**0.00028**	**0.004**	28.7	27.6	-1.1 (-2.6 to 5)	0.48	1	6.1 (-12 to 1.3)	0.12	0.36

**PC 18:2;0_18:3;0**	2.6	1.6	-0.97 (0.43 to 1.9)	**0.00033**	**0.0045**	2.2	2.1	-0.042 (-0.54 to 0.42)	0.75	1	1.5 (-2.4 to -0.25)	**0.011**	0.17
**Cer 40:1;2**	1.5	1.3	-0.2 (0.084 to 0.29)	**0.00038**	**0.005**	1.5	1.4	-0.063 (-0.031 to 0.18)	0.16	1	0.14 (-0.28 to 0.08)	0.27	0.49
**PE O-18:1;0/20:3;0**	0.69	0.54	-0.15 (0.07 to 0.26)	**0.00042**	**0.0053**	0.71	0.59	-0.12 (0.006 to 0.19)	**0.036**	0.97	0.098 (-0.26 to 0.035)	0.14	0.37
**PC 18:2;0_20:4;0**	11.6	10	-1.5 (0.46 to 2.1)	**0.00067**	**0.0077**	11.5	11.9	0.43 (-1.3 to 0.69)	0.56	1	2 (-3.2 to 0.11)	0.088	0.33
**PC 16:1;0_18:0;0**	2.4	1.9	-0.46 (0.16 to 0.62)	**0.00067**	**0.0077**	2.3	2.3	-0.035 (-0.16 to 0.32)	0.49	1	0.42 (-0.79 to 0.13)	0.16	0.37
**SM 40:1;2**	31.3	27.9	-3.4 (1.6 to 4.9)	**0.0008**	**0.0087**	30.8	31.2	0.44 (-2.5 to 2.2)	0.98	1	3.9 (-6.8 to 0.17)	0.055	0.31
**PE O-16:1;0/18:2;0**	1.6	1.3	-0.32 (0.15 to 0.57)	**0.00081**	**0.0087**	1.5	1.4	-0.095 (-0.11 to 0.29)	0.26	1	0.32 (-0.72 to 0.073)	0.096	0.34
**PE O-18:2;0/18:1;0**	0.59	0.46	-0.12 (0.05 to 0.19)	**0.00087**	**0.009**	0.58	0.52	-0.065 (0.0069 to 0.12)	**0.025**	0.92	0.062 (-0.14 to 0.049)	0.31	0.52
**PC 18:1;0_18:3;0**	3.1	2	-1.1 (0.36 to 1.4)	**0.00094**	**0.0092**	2.7	2.8	0.12 (-0.49 to 0.39)	1	1	1.3 (-1.8 to -0.26)	**0.0034**	0.17
**PC O-18:1;0/20:3;0**	1.2	0.98	-0.21 (0.079 to 0.34)	**0.00095**	**0.0092**	1.2	1.1	-0.13 (-0.049 to 0.29)	0.12	1	0.095 (-0.27 to 0.036)	0.11	0.35
**PE 16:0;0_20:3;0**	0.47	0.39	-0.079 (0.042 to 0.13)	**0.001**	**0.0094**	0.45	0.46	0.01 (-0.048 to 0.062)	0.8	1	0.13 (-0.24 to -0.025)	**0.011**	0.17
**PI 18:1;0_18:1;0**	0.97	0.65	-0.31 (0.11 to 0.45)	**0.001**	**0.0094**	0.88	0.85	-0.033 (-0.092 to 0.17)	0.66	1	0.29 (-0.53 to -0.065)	**0.016**	0.22

**PC O-16:2;0/18:1;0**	0.37	0.3	-0.068 (0.034 to 0.12)	**0.0013**	**0.011**	0.33	0.35	0.019 (-0.056 to 0.027)	0.56	1	0.085 (-0.16 to -0.026)	**0.011**	0.17
**LPE 20:0;0**	0.5	0.41	-0.088 (0.02 to 0.11)	**0.0013**	**0.011**	0.5	0.48	-0.021 (-0.025 to 0.07)	0.42	1	0.089 (-0.16 to -0.0095)	**0.022**	0.22
**PE O-16:1;0/18:1;0**	0.53	0.44	-0.091 (0.042 to 0.17)	**0.0013**	**0.011**	0.55	0.51	-0.042 (-0.014 to 0.1)	0.11	1	0.071 (-0.15 to 0.042)	0.35	0.55
**PC O-18:2;0/20:3;0**	0.59	0.46	-0.13 (0.048 to 0.2)	**0.0015**	**0.011**	0.58	0.49	-0.084 (-0.061 to 0.13)	0.26	1	0.086 (-0.16 to 0.0095)	0.15	0.37
**PC 18:1;0_18:2;0**	64.4	54.2	-10 (4 to 16)	**0.0015**	**0.011**	63.3	62.4	-0.9 (-4.3 to 5.4)	0.87	1	9.2 (-20 to 2.1)	0.15	0.37
**PE O-16:1;0/22:5;0**	2	1.6	-0.35 (0.13 to 0.46)	**0.0015**	**0.011**	2	1.9	-0.11 (-0.099 to 0.32)	0.37	1	0.23 (-0.54 to 0.17)	0.37	0.57
**PC 18:0;0_20:5;0**	10.3	8	-2.3 (0.99 to 3.5)	**0.0016**	**0.012**	9.7	10.4	0.64 (-1.9 to 1)	0.72	1	2.8 (-5.3 to -0.5)	**0.021**	0.22
**PC O-16:0;0/18:3;0**	0.31	0.23	-0.077 (0.021 to 0.16)	**0.0017**	**0.012**	0.27	0.28	0.0011 (-0.055 to 0.049)	0.99	1	0.21 (-0.37 to -0.042)	**0.0063**	0.17
**SM 38:1;2**	17.8	16.1	-1.7 (0.61 to 2.6)	**0.0017**	**0.012**	17.3	17.7	0.41 (-1.5 to 0.97)	0.65	1	2.1 (-3.6 to -0.16)	**0.029**	0.24
**Cer 42:1;2**	3.8	3.3	-0.49 (0.2 to 0.76)	**0.0017**	**0.012**	3.7	3.7	-0.077 (-0.097 to 0.43)	0.14	1	0.42 (-0.75 to 0.17)	0.27	0.49
**PC 17:0;0_18:2;0**	16.3	14.5	-1.8 (0.48 to 2.9)	**0.0019**	**0.012**	16.4	16.7	0.3 (-2.3 to 0.96)	0.41	1	2.1 (-4.5 to 0.45)	0.11	0.35
**PE O-18:2;0/16:0;0**	0.21	0.18	-0.03 (0.015 to 0.06)	**0.0019**	**0.012**	0.21	0.2	-0.01 (-0.014 to 0.044)	0.32	1	0.025 (-0.056 to 0.02)	0.37	0.57
**LPE 18:2;0**	1.2	0.91	-0.29 (0.084 to 0.44)	**0.002**	**0.013**	1.2	1.1	-0.068 (-0.12 to 0.21)	0.52	1	0.23 (-0.45 to 0.016)	0.07	0.33

**PC O-18:0;0/18:3;0**	0.17	0.11	-0.055 (0.015 to 0.074)	**0.002**	**0.013**	0.14	0.14	0.0042 (-0.023 to 0.029)	0.75	1	0.067 (-0.11 to 0.0052)	0.074	0.33
**PC 17:0;0_18:3;0**	0.55	0.4	-0.14 (0.025 to 0.16)	**0.002**	**0.013**	0.79	0.95	0.16 (-0.066 to 0.069)	0.96	1	0.088 (-0.19 to 0.081)	0.46	0.65
**PC 16:0;0_20:1;0**	1.8	1.6	-0.18 (0.094 to 0.34)	**0.0022**	**0.013**	1.8	1.8	0.014 (-0.16 to 0.14)	0.92	1	0.2 (-0.4 to -0.054)	**0.02**	0.22
**PE 18:1;0_20:3;0**	0.83	0.68	-0.15 (0.072 to 0.22)	**0.0023**	**0.014**	0.81	0.82	0.0051 (-0.11 to 0.11)	0.94	1	0.13 (-0.3 to 0.035)	0.13	0.37
**PC O-18:2;0/18:2;0**	2.7	2.2	-0.45 (0.15 to 0.63)	**0.0026**	**0.015**	2.6	2.6	-0.023 (-0.3 to 0.37)	0.66	1	0.44 (-0.85 to 0.12)	0.1	0.35
**PC O-17:2;0/17:0;0**	0.16	0.13	-0.024 (0.0088 to 0.039)	**0.0029**	**0.017**	0.16	0.16	-0.0056 (-0.01 to 0.026)	0.32	1	0.012 (-0.037 to 0.015)	0.37	0.57
**LPC 18:2;0**	17.2	13.1	-4.1 (0.84 to 4.6)	**0.003**	**0.017**	16.5	16.9	0.48 (-2.5 to 2)	0.93	1	4.8 (-7.3 to -1.1)	**0.015**	0.22
**PC O-16:1;0/18:2;0**	6.9	5.8	-1.1 (0.36 to 1.7)	**0.003**	**0.017**	6.5	6.5	-0.02 (-0.63 to 0.7)	0.88	1	1.2 (-2.2 to -0.033)	**0.039**	0.25
**PC 18:2;0_20:0;0**	0.84	0.68	-0.16 (0.06 to 0.29)	**0.0032**	**0.017**	0.76	0.79	0.032 (-0.079 to 0.1)	0.6	1	0.26 (-0.4 to -0.081)	**0.0043**	0.17
**SM 32:1;2**	11.9	10.8	-1.1 (0.34 to 1.8)	**0.0032**	**0.017**	11.8	12	0.18 (-1.1 to 0.7)	0.85	1	1.4 (-2.5 to 0.076)	0.067	0.33
**CE 20:3;0**	45.2	38.4	-6.7 (2.3 to 11)	**0.0035**	**0.018**	46.5	44.9	-1.6 (-2 to 6.4)	0.26	1	5.1 (-12 to 1.3)	0.15	0.37
**PC 18:2;0_20:2;0**	2.2	1.8	-0.32 (0.15 to 0.58)	**0.0037**	**0.019**	2.2	2.1	-0.16 (-0.11 to 0.43)	0.21	1	0.23 (-0.74 to 0.24)	0.28	0.49

**PC 14:0;0_20:4;0**	2.8	2.5	-0.32 (0.16 to 0.91)	**0.0039**	**0.02**	2.8	2.9	0.099 (-0.33 to 0.27)	0.86	1	0.71 (-1.2 to -0.27)	**0.006**	0.17
**PC 16:0;0_20:2;0**	8.5	7.5	-1 (0.3 to 1.8)	**0.0043**	**0.022**	8.7	8.2	-0.55 (-0.11 to 1.4)	0.066	1	0.55 (-1.6 to 0.63)	0.53	0.69
**PI 18:0;0_18:1;0**	2.2	1.7	-0.41 (0.14 to 0.63)	**0.0047**	**0.023**	2.3	2.1	-0.15 (-0.11 to 0.42)	0.31	1	0.27 (-0.61 to 0.09)	0.18	0.4
**PC O-16:2;0/16:0;0**	2.8	2.2	-0.54 (0.13 to 0.9)	**0.005**	**0.024**	2.6	2.7	0.074 (-0.27 to 0.33)	0.81	1	0.69 (-1.2 to -0.094)	**0.025**	0.24
**PI 16:0;0_18:1;0**	1	0.75	-0.3 (0.096 to 0.45)	**0.005**	**0.024**	1.1	1.1	0.021 (-0.25 to 0.19)	0.82	1	0.34 (-0.59 to -0.07)	**0.026**	0.24
**PI 18:0;0_20:3;0**	2.6	2.2	-0.32 (0.1 to 0.52)	**0.005**	**0.024**	2.8	2.6	-0.21 (-0.048 to 0.53)	0.09	1	0.1 (-0.48 to 0.28)	0.53	0.69
**CE 17:1;0**	12.8	11	-1.8 (0.53 to 3)	**0.0054**	**0.025**	13.1	13.1	-0.024 (-1.7 to 1.5)	0.82	1	1.8 (-4.1 to 0.55)	0.12	0.35
**PE O-18:2;0/22:5;0**	1.8	1.5	-0.34 (0.099 to 0.62)	**0.0056**	**0.025**	1.9	1.6	-0.25 (0.028 to 0.53)	**0.033**	0.97	0.1 (-0.48 to 0.37)	0.94	0.97
**PC 14:0;0_18:1;0**	4.4	3.6	-0.86 (0.27 to 1.4)	**0.0058**	**0.025**	4.5	4.5	0.044 (-0.52 to 0.45)	0.79	1	0.9 (-1.5 to -0.18)	**0.011**	0.17
**LPC 18:1;0**	13.3	10.8	-2.5 (0.4 to 3)	**0.0058**	**0.025**	13	13.3	0.24 (-1.6 to 1.5)	0.96	1	2.9 (-4.6 to -0.48)	**0.011**	0.17
**PC O-18:0;0/20:5;0**	0.86	0.75	-0.11 (0.049 to 0.24)	**0.0059**	**0.026**	0.87	0.93	0.056 (-0.16 to 0.078)	0.45	1	0.18 (-0.35 to -0.053)	**0.007**	0.17
**PC 18:1;0_20:5;0**	6.5	5.4	-1.1 (0.35 to 2)	**0.0061**	**0.026**	6.5	6.6	0.099 (-0.99 to 0.74)	0.9	1	1.2 (-3 to 0.44)	0.14	0.37

**PC O-16:2;0/18:0;0**	0.32	0.25	-0.069 (0.015 to 0.1)	**0.0062**	**0.026**	0.32	0.31	-0.019 (-0.032 to 0.062)	0.57	1	0.058 (-0.11 to -0.01)	**0.02**	0.22
**PC 14:0;0_20:3;0**	0.82	0.7	-0.12 (0.046 to 0.33)	**0.0066**	**0.027**	0.79	0.81	0.015 (-0.12 to 0.2)	0.7	1	0.17 (-0.37 to 0.026)	0.098	0.34
**PC O-17:0;0/17:1;0**	0.44	0.38	-0.069 (0.017 to 0.11)	**0.0066**	**0.027**	0.46	0.45	-0.014 (-0.049 to 0.068)	0.84	1	0.063 (-0.12 to 0.019)	0.18	0.39
**CE 14:0;0**	26.7	22	-4.6 (1.6 to 9.2)	**0.008**	**0.031**	27	26.8	-0.12 (-2.8 to 3.3)	0.98	1	5.8 (-11 to -1)	**0.018**	0.22
**PC O-16:0;0/18:1;0**	2.7	2.4	-0.28 (0.068 to 0.44)	**0.0081**	**0.031**	2.7	2.7	0.068 (-0.33 to 0.25)	0.98	1	0.38 (-0.61 to 0.025)	0.07	0.33
**PC O-16:0;0/16:0;0**	7.4	6.7	-0.71 (0.2 to 1.2)	**0.0081**	**0.031**	7.8	7.6	-0.17 (-0.37 to 0.96)	0.42	1	0.65 (-1.5 to 0.28)	0.23	0.45
**PC 18:1;0_20:3;0**	11.9	10.6	-1.3 (0.41 to 2.2)	**0.0081**	**0.031**	12.3	11.9	-0.47 (-0.34 to 1.5)	0.18	1	0.83 (-2.1 to 0.63)	0.3	0.51
**CE 18:2;0**	2741.2	2489.3	-250 (74 to 440)	**0.0081**	**0.031**	2756.7	2744.5	-12 (-200 to 250)	0.68	1	240 (-660 to 160)	0.3	0.51
**PC 16:0;0_20:3;0**	66.6	59.1	-7.5 (1.8 to 12)	**0.0081**	**0.031**	69	63.9	-5.2 (-0.34 to 12)	0.069	1	2.4 (-10 to 6.7)	0.7	0.79
**PI 18:1;0_20:3;0**	0.34	0.28	-0.064 (0.017 to 0.12)	**0.0083**	**0.031**	0.32	0.31	-0.012 (-0.035 to 0.073)	0.68	1	0.076 (-0.14 to 0.01)	0.083	0.33
**PC O-16:1;0/20:3;0**	1.5	1.2	-0.28 (0.074 to 0.44)	**0.0087**	**0.032**	1.5	1.5	-0.032 (-0.18 to 0.19)	0.96	1	0.25 (-0.57 to 0.051)	0.11	0.35

**CE 16:1;0**	150.1	129.8	-20 (5.7 to 30)	**0.0087**	**0.032**	156	156.1	0.072 (-11 to 16)	0.76	1	21 (-43 to 6)	0.14	0.37
**PC O-16:0;0/18:0;0**	0.12	0.11	-0.013 (0.0038 to 0.051)	**0.009**	**0.033**	0.14	0.11	-0.026 (-0.002 to 0.019)	0.09	1	0.0079 (-0.037 to 0.016)	0.49	0.67
**PE O-16:1;0/20:3;0**	0.39	0.31	-0.084 (0.02 to 0.15)	**0.0092**	**0.033**	0.41	0.43	0.012 (-0.047 to 0.068)	0.56	1	0.088 (-0.2 to 0.0085)	0.096	0.34
**LPC 18:0;0**	18.4	15.5	-2.9 (0.44 to 3.9)	**0.0093**	**0.033**	19.3	18.4	-0.98 (-1.1 to 3)	0.37	1	2.2 (-4.4 to 0.075)	0.058	0.32
**PC O-16:0;0/20:3;0**	1.4	1.2	-0.2 (0.064 to 0.45)	**0.0095**	**0.033**	1.5	1.4	-0.11 (-0.026 to 0.29)	0.079	1	0.18 (-0.45 to -0.021)	**0.034**	0.24
**PC 17:1;0_18:2;0**	3	2.7	-0.25 (0.073 to 0.88)	**0.0096**	**0.033**	3.1	2.8	-0.23 (-0.17 to 0.51)	0.44	1	0.56 (-0.79 to 0.072)	0.15	0.37
**PC 17:0;0_20:5;0**	1	0.68	-0.35 (0.031 to 0.54)	**0.01**	**0.034**	0.89	1.4	0.48 (-0.21 to 0.048)	0.41	1	0.66 (-0.76 to -0.026)	**0.02**	0.22
**PE O-18:0;0/18:2;0**	0.29	0.25	-0.04 (0.013 to 0.094)	**0.01**	**0.035**	0.29	0.27	-0.015 (-0.015 to 0.055)	0.23	1	0.035 (-0.089 to 0.019)	0.27	0.49
**PE 18:0;0_20:5;0**	0.59	0.47	-0.12 (0.011 to 0.2)	**0.011**	**0.035**	0.58	0.61	0.024 (-0.066 to 0.1)	0.79	1	0.052 (-0.18 to 0.076)	0.24	0.47
**PC O-16:1;0/22:5;0**	1	0.87	-0.13 (0.037 to 0.28)	**0.012**	**0.039**	0.93	0.98	0.052 (-0.25 to 0.085)	0.43	1	0.34 (-0.56 to -0.091)	**0.0096**	0.17
**PE 18:1;0_18:1;0**	0.9	0.75	-0.16 (0.028 to 0.26)	**0.012**	**0.039**	0.88	0.94	0.056 (-0.18 to 0.068)	0.39	1	0.22 (-0.35 to -0.018)	**0.028**	0.24

**PC 18:1;0_18:1;0**	24.7	21	-3.7 (0.67 to 6.1)	**0.014**	**0.044**	23.6	24.4	0.78 (-2.7 to 1.2)	0.47	1	4.5 (-8.1 to -0.17)	**0.036**	0.24
**PC 16:1;0_20:3;0**	1.8	1.7	-0.15 (0.044 to 0.39)	**0.014**	**0.044**	1.9	1.8	-0.063 (-0.059 to 0.3)	0.25	1	0.17 (-0.51 to 0.19)	0.34	0.54
**PC O-16:1;0/18:0;0**	0.44	0.39	-0.05 (0.009 to 0.089)	**0.015**	**0.046**	0.43	0.44	0.0094 (-0.048 to 0.047)	0.72	1	0.062 (-0.12 to -0.0032)	**0.035**	0.24
**PI 16:1;0_18:0;0**	1.1	0.98	-0.098 (0.02 to 0.17)	**0.015**	**0.046**	1.1	1.1	-0.014 (-0.073 to 0.11)	0.52	1	0.087 (-0.21 to 0.014)	0.081	0.33
**PC O-16:0;0/18:2;0**	5.2	4.4	-0.84 (0.15 to 1.4)	**0.015**	**0.046**	5.3	5.2	-0.094 (-0.6 to 0.77)	0.68	1	0.78 (-1.9 to 0.24)	0.12	0.35
**PE O-18:2;0/18:2;0**	1.3	1.1	-0.25 (0.059 to 0.45)	**0.015**	**0.046**	1.3	1.2	-0.082 (-0.13 to 0.29)	0.43	1	0.24 (-0.57 to 0.11)	0.19	0.4
**PI 16:0;0_20:4;0**	2	1.7	-0.27 (0.05 to 0.44)	**0.015**	**0.046**	2	2	0.0083 (-0.19 to 0.23)	0.82	1	0.29 (-0.52 to 0.1)	0.21	0.43
**PC 16:0;0_18:2;0**	466	420	-46 (12 to 70)	**0.015**	**0.046**	470	461	-8.9 (-25 to 45)	0.61	1	38 (-100 to 43)	0.64	0.77
**PC 18:2;0_20:1;0**	2	1.7	-0.36 (0.084 to 0.67)	**0.016**	**0.048**	1.8	1.9	0.11 (-0.34 to 0.11)	0.28	1	0.47 (-0.93 to -0.034)	**0.032**	0.24
**CE 18:1;0**	798	720	-78 (19 to 130)	**0.016**	**0.048**	813	833	20 (-78 to 57)	0.78	1	97 (-200 to 28)	0.15	0.37
**PI 16:0;0_20:3;0**	0.46	0.4	-0.062 (0.015 to 0.11)	**0.017**	**0.049**	0.47	0.45	-0.023 (-0.014 to 0.065)	0.18	1	0.053 (-0.13 to 0.02)	0.16	0.38
**TAG 48:0;0**	6.8	4.4	-2.4 (0.27 to 3.1)	**0.018**	0.052	7	6.7	-0.24 (-0.93 to 2.3)	0.24	1	1.8 (-3.7 to 1.4)	0.5	0.67

**Cer 40:0;2**	0.27	0.24	-0.031 (0.0045 to 0.078)	**0.019**	0.055	0.28	0.3	0.019 (-0.045 to 0.018)	0.57	1	0.082 (-0.14 to -0.025)	**0.0052**	0.17
**PC 18:2;0_19:0;0**	1.4	1.2	-0.18 (0.023 to 0.37)	**0.019**	0.055	1.3	1.4	0.069 (-0.31 to 0.071)	0.23	1	0.43 (-0.76 to -0.15)	**0.0095**	0.17
**PC O-16:1;0/18:1;0**	1.2	1	-0.17 (0.023 to 0.24)	**0.02**	0.055	1.2	1.2	0.0036 (-0.17 to 0.14)	0.9	1	0.18 (-0.35 to 0.048)	0.15	0.37
**PE O-18:2;0/20:3;0**	0.71	0.53	-0.18 (0.021 to 0.25)	**0.021**	0.059	0.73	0.61	-0.11 (0.0011 to 0.21)	**0.048**	1	0.066 (-0.3 to 0.16)	0.95	0.98
**PC O-17:0;0/17:0;0**	0.54	0.5	-0.039 (0.0047 to 0.12)	**0.022**	0.059	0.56	0.54	-0.014 (-0.064 to 0.055)	0.79	1	0.064 (-0.18 to 0.012)	0.096	0.34
**PC 18:0;0_22:4;0**	2.4	2.2	-0.22 (0.035 to 0.54)	**0.023**	0.063	2.4	2.4	0.006 (-0.16 to 0.17)	0.64	1	0.32 (-0.65 to -0.07)	**0.02**	0.22
**PE 18:1;0_18:2;0**	0.95	0.77	-0.19 (0.028 to 0.36)	**0.023**	0.063	0.94	1	0.071 (-0.21 to 0.079)	0.48	1	0.26 (-0.51 to 0.024)	0.096	0.34
**PE 18:0;0_18:2;0**	10.8	9.7	-1.1 (0.15 to 1.8)	**0.023**	0.063	11.1	10.7	-0.44 (-0.44 to 1.4)	0.35	1	0.66 (-2.2 to 0.97)	0.43	0.63
**PC O-18:1;0/16:0;0**	1.6	1.5	-0.16 (0.02 to 0.24)	**0.025**	0.066	1.6	1.6	0.0041 (-0.16 to 0.19)	0.56	1	0.17 (-0.3 to 0.094)	0.49	0.67
**PC 14:0;0_18:2;0**	16.5	14.1	-2.4 (0.49 to 4.5)	**0.026**	0.068	16.6	15.8	-0.78 (-1 to 3)	0.37	1	1.7 (-4.3 to 0.49)	0.088	0.33
**TAG 56:5;0**	8.2	9.2	1.1 (-1.9 to -0.068)	**0.026**	0.068	8.7	8.3	-0.43 (-0.88 to 1.8)	0.43	1	-0.91 (-0.63 to 2.7)	0.16	0.37

**PI 18:1;0_18:2;0**	0.57	0.48	-0.097 (0.011 to 0.21)	**0.026**	0.068	0.56	0.56	-7.7e-05 (-0.065 to 0.093)	0.67	1	0.13 (-0.3 to 0.06)	0.21	0.43
**Cer 42:0;2**	0.33	0.28	-0.041 (0.0042 to 0.091)	**0.027**	0.07	0.33	0.33	-0.004 (-0.028 to 0.045)	0.75	1	0.054 (-0.1 to 0.013)	0.18	0.39
**PC 15:0;0_18:2;0**	16.5	14.5	-2 (0.13 to 3)	**0.028**	0.071	16.1	16.2	0.088 (-1.7 to 1.5)	0.61	1	2 (-4.4 to 0.9)	0.21	0.42
**PC 17:1;0_18:1;0**	2.3	2.1	-0.29 (0.02 to 0.45)	**0.029**	0.073	2.3	2.4	0.084 (-0.19 to 0.12)	0.67	1	0.24 (-0.58 to 0.12)	0.21	0.43
**PC O-18:1;0/18:2;0**	2.5	2.1	-0.32 (0.045 to 0.61)	**0.03**	0.075	2.5	2.4	-0.1 (-0.32 to 0.38)	0.56	1	0.37 (-0.85 to 0.015)	0.06	0.32
**PC O-16:0;0/20:5;0**	0.71	0.58	-0.13 (0.015 to 0.28)	**0.03**	0.075	0.69	0.75	0.058 (-0.16 to 0.15)	0.89	1	0.15 (-0.46 to 0.15)	0.26	0.48
**PC 18:1;0_20:1;0**	0.65	0.59	-0.055 (0.0073 to 0.18)	**0.031**	0.076	0.63	0.67	0.043 (-0.11 to 0.082)	0.66	1	0.16 (-0.34 to -0.0094)	**0.033**	0.24
**PC 16:0;0_20:5;0**	24.6	21	-3.6 (0.25 to 6.8)	**0.031**	0.076	23.7	25.2	1.5 (-4.8 to 2.8)	0.79	1	4.8 (-11 to 0.91)	0.088	0.33
**PC O-16:1;0/16:0;0**	4.3	3.8	-0.42 (0.025 to 0.69)	**0.033**	0.079	4.1	4.2	0.06 (-0.35 to 0.42)	0.82	1	0.48 (-0.99 to 0.073)	0.08	0.33
**PE 16:0;0_18:2;0**	2	1.8	-0.18 (0.022 to 0.4)	**0.036**	0.087	2.1	2	-0.12 (-0.14 to 0.32)	0.56	1	0.067 (-0.43 to 0.23)	0.58	0.72
**PC 16:0;0_19:1;0**	1.2	1.1	-0.12 (0.013 to 0.25)	**0.037**	0.087	1.3	1.3	-0.01 (-0.16 to 0.14)	0.99	1	0.12 (-0.31 to 0.092)	0.26	0.48

**TAG 50:5;0**	3	2.4	-0.61 (0.047 to 1.1)	**0.038**	0.09	2.6	3.2	0.62 (-0.66 to 0.43)	0.85	1	1.5 (-1.6 to -0.07)	**0.02**	0.22
**CE 18:0;0**	18.6	16.4	-2.2 (0.13 to 4.1)	**0.038**	0.09	19.7	19.5	-0.24 (-2 to 2)	0.93	1	2 (-4.6 to 0.91)	0.18	0.39
**PC 17:0;0_22:4;0**	1.6	1.4	-0.22 (0.0076 to 0.35)	**0.039**	0.09	1.4	1.6	0.21 (-0.55 to 0.06)	0.14	1	0.51 (-1.1 to 0.11)	0.12	0.36
**PC 16:0;0_22:6;0**	62.6	68.9	6.2 (-11 to -0.42)	**0.04**	0.093	64.6	68.7	4.1 (-13 to 4.8)	0.46	1	-2.1 (-8 to 12)	0.58	0.72
**PC O-16:0;0/22:4;0**	0.61	0.56	-0.05 (0.0031 to 0.11)	**0.041**	0.093	0.61	0.58	-0.031 (-0.068 to 0.11)	0.61	1	0.028 (-0.15 to 0.096)	0.69	0.79
**PC 17:1;0_18:0;0**	0.64	0.54	-0.098 (0.0047 to 0.16)	**0.043**	0.097	0.64	0.65	0.011 (-0.12 to 0.059)	0.43	1	0.14 (-0.23 to 0.013)	0.064	0.33
**TAG 52:3;0**	202.8	223.1	20 (-49 to -1.6)	**0.043**	0.096	222.4	218.1	-4.2 (-26 to 37)	0.69	1	-25 (-7.8 to 71)	0.11	0.35
**PE O-18:0;0/20:4;0**	0.6	0.52	-0.082 (0.0033 to 0.19)	**0.043**	0.096	0.63	0.56	-0.069 (-0.022 to 0.13)	0.21	1	0.079 (-0.21 to 0.076)	0.37	0.57
**Cer 40:2;2**	0.37	0.36	-0.012 (0.00091 to 0.056)	**0.044**	0.097	0.35	0.38	0.025 (-0.04 to 0.0092)	0.27	1	0.042 (-0.083 to -0.016)	**0.012**	0.18
**PC 17:0;0_20:6;0**	2.3	2.2	-0.081 (0.01 to 0.56)	**0.045**	0.098	2.3	2.2	-0.043 (-0.36 to 0.34)	0.95	1	0.41 (-0.85 to 0.14)	0.15	0.37
**PC 14:0;0_16:0;0**	3.6	3.1	-0.52 (0.0027 to 1.3)	**0.046**	0.099	3.8	3.8	0.00088 (-0.61 to 0.41)	0.66	1	0.68 (-1.5 to 0.12)	0.076	0.33
**CE 20:2;0**	5.8	5.2	-0.57 (0.11 to 1.4)	**0.046**	0.099	7.2	5.7	-1.5 (-0.66 to 1.1)	0.62	1	0.15 (-2.1 to 0.4)	0.14	0.37
**SM 34:0;2**	3.7	3.4	-0.38 (0.0063 to 0.68)	**0.047**	0.1	3.7	4	0.22 (-0.53 to 0.11)	0.16	1	0.61 (-1.1 to -0.036)	**0.038**	0.25

**PC O-18:2;0/18:1;0**	0.62	0.55	-0.074 (-0.0039 to 0.16)	0.06	0.12	0.61	0.61	0.008 (-0.095 to 0.079)	0.95	1	0.11 (-0.22 to -0.0054)	**0.034**	0.24
**TAG 52:6;0**	5.7	4.2	-1.5 (-0.084 to 1.6)	0.063	0.13	4.3	5.6	1.2 (-1 to 0.63)	0.71	1	3 (-2.4 to -0.016)	**0.045**	0.28
**LPE 20:1;0**	0.39	0.31	-0.074 (-0.004 to 0.13)	0.08	0.15	0.33	0.36	0.032 (-0.079 to 0.032)	0.54	1	0.16 (-0.25 to -0.069)	**0.0024**	0.17
**PC O-17:1;0/17:0;0**	0.12	0.1	-0.013 (-0.0015 to 0.027)	0.088	0.16	0.11	0.11	-0.0052 (-0.005 to 0.01)	0.59	1	0.011 (-0.029 to -0.00036)	**0.048**	0.29
**PE O-16:2;0/18:0;0**	0.074	0.062	-0.012 (-0.0023 to 0.021)	0.12	0.2	0.079	0.07	-0.0097 (4e-04 to 0.02)	**0.041**	0.97	0.00017 (-0.022 to 0.018)	0.95	0.97
**LPE 16:0;0**	0.71	0.63	-0.076 (-0.012 to 0.13)	0.13	0.21	0.69	0.72	0.027 (-0.1 to 0.057)	0.69	1	0.12 (-0.24 to -0.017)	**0.029**	0.24
**PC O-18:0;0/20:6;0**	1.1	1	-0.079 (-0.098 to 0.24)	0.36	0.46	1.2	1.1	-0.1 (0.0026 to 0.3)	**0.042**	0.97	-0.02 (-0.27 to 0.31)	0.89	0.94
**PC 17:0;0_20:4;0**	7.8	7.9	0.12 (-0.82 to 0.62)	0.63	0.72	7.9	8.4	0.51 (-1.3 to -0.084)	**0.018**	0.85	0.41 (-1.3 to 0.58)	0.56	0.71
**SM 34:1;3**	1	1	0.031 (-0.12 to 0.16)	0.78	0.83	0.89	1.1	0.16 (-0.3 to -0.084)	**0.0013**	0.39	0.26 (-0.56 to 0.017)	0.067	0.33
**PC 18:1;0_20:4;0**	19.9	19.6	-0.34 (-1.6 to 1.9)	1	1	20.1	21.7	1.6 (-2.5 to -0.068)	**0.039**	0.97	1.9 (-3.6 to 1.1)	0.37	0.57

Lipid species showing significant changes within eTRE, lTRE, and/or between interventions are shown. Data are shown as mean or mean (95% CI).

[a)] P-values without correction for multiple testing.

[b)] P-values with correction for multiple testing.

Table S6. Lipid classes showing alterations within or between eTRE and lTRE interventions.

**Lipid classes**	**Before eTRE**	**After eTRE**	**Change eTRE after – before (95% CI)**	**P[a)]**	**P BH[b)]**	**Before lTRE**	**After lTRE**	**Change lTRE After – before (95% CI)**	**P[a)]**	**P BH[b)]**	**Difference between lTRE vs. eTRE (95% CI)[a]**	**P[a)]**	**P BH[b)]**
Cer	8.5	7.6	-0.88 (0.26 to 1.4)	**0.0054**	**0.043**	8.3	8.2	-0.11 (-0.39 to 0.75)	0.3	0.99	0.78 (-1.5 to 0.43)	0.31	0.62
PC	1894.6	1711.8	-180 (43 to 260)	**0.0062**	**0.043**	1911.7	1909.9	-1.8 (-110 to 140)	0.61	0.99	180 (-380 to 94)	0.48	0.67
PE O-	52.3	44.4	-7.9 (2.1 to 12)	**0.013**	0.06	52.8	50.5	-2.2 (-3.2 to 7.2)	0.58	0.99	6.1 (-14 to 4.3)	0.29	0.62
CE	4870	4453.8	-420 (76 to 740)	**0.021**	0.061	4940.1	5004.1	64 (-420 to 390)	0.9	0.99	480 (-1200 to 280)	0.3	0.62
LPC	105	90	-15 (1.4 to 18)	**0.023**	0.061	107.3	106.5	-0.77 (-11 to 13)	0.93	0.99	16 (-28 to -0.67)	0.04	0.28
LPE	8.1	6.9	-1.3 (0.13 to 1.9)	**0.026**	0.061	7.9	8	0.12 (-0.94 to 0.88)	0.99	0.99	1.5 (-2.4 to -0.27)	**0.015**	0.22
PC O-	96.2	86.6	-9.6 (0.81 to 18)	**0.036**	0.073	97.6	98.3	0.74 (-10 to 9.2)	0.96	0.99	11 (-24 to 2.3)	0.1	0.47

Data are shown as mean or mean (95% CI).

a) P-values without correction for multiple testing.

b) P-values with correction for multiple testing.

Table S7. Fatty acids showing alterations within or between eTRE and lTRE interventions.

Fatty acid	Before eTRE	After eTRE	Change eTRE after – before (95% CI)	P[a)]	P BH[b)]	Before lTRE	After lTRE	Change lTRE After – before (95% CI)	P[a)]	P BH[b)]	Difference between lTRE vs.	P[a)]	P BH[b)]
18:0;0	633.2	550.2	-83 (35 to 120)	**0.00021**	**0.010**	638.3	632.5	-5.8 (-32 to 54)	0.62	1	78 (-140 to 8.6)	0.088	0.51
20:3;0	206.8	176.5	-30 (14 to 43)	**0.00061**	**0.012**	211.9	199.9	-12 (-1.9 to 32)	0.079	1	18 (-41 to 7.6)	0.16	0.51
20:2;0	30.9	26.4	-4.5 (2.3 to 6.9)	**0.00073**	**0.012**	32.1	29.6	-2.5 (-1.3 to 6.1)	0.17	1	2 (-6.3 to 2.8)	0.4	0.64
O-17:2;0	0.16	0.13	-0.024 (0.0088 to 0.039)	**0.0029**	**0.026**	0.16	0.16	-0.0056 (-0.01 to 0.026)	0.32	1	0.012 (-0.037 to 0.015)	0.37	0.63
18:3;0	242.4	182.4	-60 (14 to 76)	**0.003**	**0.026**	227	240.7	14 (-21 to 35)	0.98	1	74 (-90 to 13)	0.16	0.51
O-16:2;0	3.4	2.7	-0.74 (0.22 to 1.1)	**0.0032**	**0.026**	3.2	3.4	0.18 (-0.42 to 0.36)	0.96	1	0.98 (-1.5 to -0.22)	**0.005**	0.22
20:0;0	2	1.7	-0.33 (0.099 to 0.6)	**0.008**	0.05	1.9	2.2	0.35 (-0.47 to 0.23)	0.57	1	0.81 (-1 to -0.11)	**0.018**	0.39
O-18:2;0	19.4	16.2	-3.3 (0.77 to 4.5)	**0.0087**	0.05	19.5	18.6	-0.82 (-1.5 to 3.1)	0.48	1	2.6 (-5.4 to 1.4)	0.16	0.51
O-18:1;0	38.6	34	-4.6 (0.73 to 7.3)	**0.0093**	0.05	39.3	38.8	-0.44 (-3.3 to 3.7)	0.9	1	4.6 (-9.9 to 1.6)	0.2	0.51
14:0;0	152.2	123.8	-28 (4.9 to 49)	**0.016**	0.079	156.2	156	-0.26 (-8.9 to 24)	0.43	1	28 (-46 to -0.85)	**0.043**	0.51
O-17:0;0	1.8	1.5	-0.28 (0.033 to 0.49)	**0.023**	0.1	1.8	1.8	0.035 (-0.24 to 0.12)	0.54	1	0.33 (-0.65 to 0.029)	0.061	0.51
14:1;0	10.2	7.6	-2.6 (0.2 to 4.4)	**0.029**	0.12	10.7	10.6	-0.12 (-1.6 to 2.5)	0.66	1	2.5 (-5.1 to 1.1)	0.3	0.6
18:2;0	4238.2	3891.9	-350 (22 to 580)	**0.035**	0.12	4390.6	4268.4	-120 (-240 to 470)	0.5	1	220 (-840 to 490)	0.75	0.94
O-16:0;0	30.8	27.5	-3.3 (0.33 to 6.3)	**0.035**	0.12	31.7	31.6	-0.12 (-3.5 to 3.5)	0.66	1	3.5 (-8.1 to 0.94)	0.1	0.51

20:5;0	148.4	127.5	-21 (1.4 to 41)	**0.043**	0.13	150.8	159.3	8.6 (-32 to 17)	0.72	1	29 (-66 to 6.6)	0.088	0.51
O-16:1;0	42	37.4	-4.6 (0.28 to 8.3)	**0.043**	0.13	42	41.5	-0.48 (-3.6 to 4.6)	0.81	1	4.4 (-12 to 3)	0.33	0.6
O-17:1;0	0.12	0.1	-0.013 (-0.0015 to 0.027)	0.088	0.2	0.11	0.11	-0.0052 (-0.005 to 0.01)	0.59	1	0.011 (-0.029 to -0.00036)	**0.048**	0.51

Data are shown as mean or mean (95% CI).

a) P-values without correction for multiple testing.

b) P-values with correction for multiple testing.

Table S8. Indices of enzyme activity and their calculation.

Index	Description	Calculation
D5D/FADS1	Delta-5 Desaturase	Sum 20:4 FA* / 20:3 FA*
D6D/FADS2	Delta-6 Desaturase	Sum 18:3 FA* / 18:2 FA*
D9D/SCD1 (C16)	Delta-9 Desaturase / Stearoyl-CoA Desaturase 1 - C16	Sum 16:1 FA* / 16:0 FA*
D9D/SCD1 (C18)	Delta-9 Desaturase / Stearoyl-CoA Desaturase 1 - C18	Sum 18:1 FA* / 18:0 FA*
D9D/SCD1 (C18+16)	Delta-9 Desaturase / Stearoyl-CoA Desaturase 1 - C18+16	Sum (16:1 + 18:1) FA* / (16:0 + 18:0) FA*
ELOVL5	Elongation of Very Long Chain Fatty Acids Protein 5	Sum 20:3 FA* / 18:3 FA*
ELOVL6	Elongation of Very Long Chain Fatty Acids Protein 6	Sum 18:0 FA* / 16:0 FA *

* for all lipids in which fatty acids (FA) were measured

Table S9. Changes of enzyme activity indices within or between eTRE and lTRE interventions.

**Indices**	**Before eTRE**	**After eTRE**	**Change eTRE after – before (95% CI)**	**P a)**	**P BH b)**	**Before lTRE**	**After lTRE**	**Change lTRE After – before (95% CI)**	**P a)**	**P BH b)**	**Difference between lTRE vs. eTRE (95% CI) a**	**P a)**	**P BH b)**
ELOVL6	0.26	0.23	-0.025 (0.016 to 0.033)	**0.000006**	**0.000096**	0.25	0.25	-0.0012 (-0.0097 to 0.016)	0.62	0.82	0.023 (-0.034 to -0.0071)	**0.0054**	0.062
D5D	4.7	5.6	0.85 (-1.2 to -0.37)	**0.00034**	**0.0024**	4.7	5.5	0.82 (-1.1 to -0.41)	**0.000045**	**0.00073**	-0.0062 (-0.51 to 0.56)	0.92	0.94
SCD1/D9D (C18)	4.2	4.6	0.42 (-0.68 to -0.17)	**0.0016**	**0.0073**	4.3	4.3	0.0012 (-0.37 to 0.26)	0.65	0.82	-0.42 (0.031 to 0.83)	0.026	0.15
D6D	0.056	0.047	-0.009 (0.0013 to 0.011)	**0.015**	**0.036**	0.052	0.054	0.0014 (-0.0031 to 0.0042)	0.62	0.82	0.01 (-0.011 to 0.00087)	0.14	0.4
ELOVL5	0.96	1	0.076 (-0.18 to 0.011)	0.088	0.14	0.97	0.96	-0.015 (-0.061 to 0.082)	0.53	0.82	-0.087 (-0.053 to 0.2)	0.21	0.46
SCD1/D9D (C18+16)	0.97	0.98	0.013 (-0.055 to 0.02)	0.34	0.43	0.97	0.98	0.0063 (-0.048 to 0.026)	0.53	0.82	-0.0086 (-0.038 to 0.06)	0.7	0.85
SCD1/D9D (C16)	0.16	0.15	-0.0037 (-0.0072 to 0.014)	0.53	0.62	0.16	0.16	0.0028 (-0.0097 to 0.0047)	0.49	0.82	0.0066 (-0.018 to 0.0058)	0.3	0.53

Data are shown as mean or mean (95% CI).

a) P-values without correction for multiple testing.

b) P-values with correction for multiple testing.

Table S10. Total length of carbon chain per lipid class showing alterations within or between eTRE and lTRE interventions.

Total length of carbon chain per	Before eTRE	After eTRE	Change eTRE after – before (95% CI)	P[a)]	P BH[b)]	Before lTRE	After lTRE	Change lTRE After – before (95% CI)	P[a)]	P BH[b)]	Difference between lTRE vs. eTRE (95% CI)[a]	P[a)]	P BH[b)]
PC_C_36	35.4	34.5	-0.87 (0.48 to 1.2)	**0.00003**	**0.00095**	35.3	35.1	-0.26 (-0.15 to 0.6)	0.29	0.49	0.59 (-1.1 to -0.22)	**0.015**	0.19
PI_C_38	64.8	68.4	3.6 (-5.2 to -2.3)	**0.00003**	**0.00095**	65.7	67.3	1.6 (-2.9 to -0.26)	**0.021**	0.19	-2 (-0.045 to 4.3)	0.058	0.4
LPC_C_18	45.9	43.7	-2.2 (1.1 to 3.1)	**0.000044**	**0.00095**	45.4	45.5	0.11 (-1.2 to 1)	0.99	0.99	2.2 (-3.5 to -0.74)	**0.0032**	0.052
PI_C_36	25.3	22.8	-2.5 (1.5 to 3.6)	**0.000079**	**0.0013**	24.7	23.6	-1 (0.059 to 2.1)	**0.041**	0.28	1.5 (-3.4 to 0.092)	0.067	0.4
SM_C_40	17.6	16.8	-0.74 (0.33 to 1.1)	**0.00011**	**0.0014**	17.2	17	-0.19 (-0.21 to 0.61)	0.36	0.54	0.53 (-1.1 to 0.033)	0.073	0.4
TAG_C_52	47.3	50.4	3.1 (-4.6 to -1.7)	**0.00014**	**0.0015**	47.1	47.8	0.72 (-2 to 0.74)	0.38	0.55	-2.4 (0.29 to 4.2)	**0.029**	0.29
LPC_C_16	49.5	51.9	2.4 (-3.5 to -1.1)	**0.00019**	**0.0018**	50.1	49.5	-0.53 (-0.67 to 1.5)	0.46	0.63	-2.8 (1 to 4.4)	**0.0013**	**0.044**
SM_C_36	10.2	10.9	0.69 (-1.1 to -0.34)	**0.00026**	**0.0021**	10.5	10.4	-0.15 (-0.16 to 0.47)	0.23	0.43	-0.84 (0.3 to 1.3)	**0.00019**	**0.012**
PI_C_34	8	6.9	-1.1 (0.43 to 1.6)	**0.00067**	**0.0048**	7.8	7.4	-0.39 (-0.17 to 0.91)	0.12	0.31	0.72 (-1.5 to -0.0097)	**0.047**	0.38
CE_C_22	0.89	0.99	0.096 (-0.14 to -0.041)	**0.0012**	**0.008**	0.9	0.95	0.053 (-0.11 to 0.00064)	0.055	0.31	-0.04 (-0.035 to 0.087)	0.28	0.61
SM_C_32	4	3.8	-0.26 (0.11 to 0.41)	**0.0019**	**0.011**	3.9	3.9	-0.09 (-0.023 to 0.2)	0.098	0.31	0.17 (-0.37 to 0.031)	0.096	0.42
PC_C_38	15.5	16.3	0.76 (-1.1 to -0.3)	**0.0026**	**0.013**	15.6	16.1	0.52 (-0.89 to 0.051)	0.094	0.31	-0.27 (-0.5 to 0.86)	0.57	0.82
SM_C_38	7.5	7.3	-0.24 (0.086 to 0.41)	**0.0028**	**0.013**	7.4	7.3	-0.083 (-0.1 to 0.22)	0.52	0.69	0.15 (-0.44 to 0.073)	0.13	0.43

TAG_C_48	5.4	4.2	-1.3 (0.43 to 2)	**0.0028**	**0.013**	5.7	5.1	-0.62 (-0.12 to 1.1)	0.1	0.31	0.67 (-1.7 to 0.66)	0.5	0.78
PC O-_C_34	24.3	23.1	-1.2 (0.34 to 1.8)	**0.003**	**0.013**	23.8	23.4	-0.38 (-0.26 to 1.1)	0.3	0.49	0.8 (-1.7 to 0.072)	0.1	0.42
CE_C_20	12.1	12.9	0.88 (-1.2 to -0.23)	**0.0035**	**0.014**	12.3	13.2	0.93 (-1.4 to -0.33)	**0.0014**	**0.029**	0.075 (-0.85 to 0.83)	0.78	0.93
PE O-_C_34	5	4.4	-0.57 (0.17 to 0.83)	**0.0052**	**0.02**	4.6	4.4	-0.26 (-0.11 to 0.57)	0.22	0.43	0.39 (-0.94 to 0.26)	0.35	0.64
Cer_C_40	24.6	23.6	-1 (0.23 to 1.4)	**0.0076**	**0.026**	24	24.2	0.14 (-1.1 to 1)	0.88	0.94	1.1 (-2.1 to 0.44)	0.14	0.43
Cer_C_42	75.4	76.4	1 (-1.4 to -0.23)	**0.0076**	**0.026**	76	75.8	-0.14 (-1 to 1.1)	0.88	0.94	-1.1 (-0.44 to 2.1)	0.14	0.43
LPE_C_22	9.2	10.2	1 (-1.7 to -0.2)	**0.013**	**0.042**	8.9	9.2	0.34 (-1.1 to 0.56)	0.53	0.69	-0.75 (-0.55 to 1.9)	0.38	0.65
TAG_C_46	1.3	0.84	-0.49 (0.082 to 0.78)	**0.014**	**0.043**	1.5	1.2	-0.22 (-0.03 to 0.45)	0.079	0.31	0.26 (-0.62 to 0.38)	0.72	0.88
PC O-_C_38	26.2	27.1	0.94 (-1.9 to -0.29)	**0.015**	**0.043**	26.2	26.3	0.14 (-0.92 to 0.64)	0.64	0.77	-0.81 (-0.4 to 2)	0.1	0.42
TAG_C_49	0.9	0.75	-0.15 (0.025 to 0.23)	**0.02**	0.056	0.94	0.83	-0.11 (0.0034 to 0.2)	**0.039**	0.28	0.041 (-0.19 to 0.21)	0.92	0.98
PE_C_34	6.2	5.8	-0.38 (0.042 to 0.57)	**0.025**	0.067	6.5	6.1	-0.32 (0.012 to 0.62)	**0.043**	0.28	0.061 (-0.36 to 0.45)	0.9	0.98
PE O-_C_38	55.4	56.9	1.5 (-2.1 to -0.14)	**0.028**	0.072	56.3	56.3	-0.056 (-0.92 to 0.66)	0.82	0.91	-1.5 (-0.46 to 2.7)	0.18	0.46
PE_C_36	36.9	35.4	-1.5 (0.093 to 2.4)	**0.029**	0.073	37.1	36.2	-0.93 (-0.55 to 2.4)	0.19	0.41	0.61 (-2.2 to 1.5)	0.72	0.88
PE_C_38	46.1	47.5	1.4 (-2.8 to -0.22)	**0.038**	0.092	45.3	46.3	0.93 (-2.1 to 0.34)	0.12	0.31	-0.55 (-1.1 to 2.3)	0.5	0.78
CE_C_18	75.5	74.7	-0.87 (-0.07 to 1.4)	0.096	0.22	75.2	74.1	-1.2 (0.45 to 1.6)	**0.00076**	**0.029**	-0.36 (-0.58 to 1.4)	0.38	0.65

PC_C_37	0.76	0.81	0.045 (-0.1 to 0.0074)	0.11	0.23	0.75	0.81	0.056 (-0.094 to -0.017)	**0.0058**	0.095	0.011 (-0.066 to 0.074)	0.87	0.96
LPC_C_20	4.7	4.6	-0.1 (-0.14 to 0.44)	0.27	0.43	4.5	4.9	0.42 (-0.69 to -0.16)	**0.0014**	**0.029**	0.57 (-0.91 to -0.25)	**0.0029**	0.052
PC_C_35	1.9	1.9	0.016 (-0.075 to 0.03)	0.4	0.58	1.8	1.9	0.14 (-0.18 to -0.013)	**0.014**	0.15	0.12 (-0.21 to -0.013)	**0.031**	0.29
SM_C_42	23.1	23.3	0.24 (-0.61 to 0.24)	0.48	0.64	23.1	23.6	0.46 (-0.82 to -0.14)	**0.0097**	0.13	0.19 (-0.86 to 0.29)	0.21	0.48

Data are shown as mean or mean (95% CI).

Lipid classes & total length of carbon chain are shown as, for example, “PC_C_36” meaning all measured phosphatidylcholines with a total carbon chain length of 36 atoms.

[a] P-values without correction for multiple testing.

[b] P-values with correction for multiple testing.

Table S11. Total double bonds per lipid class showing alterations within and between eTRE and/or lTRE interventions.

**Total double bonds per lipid class**	**Before eTRE**	**After eTRE**	**Change eTRE after – before (95% CI)**	**P[a)]**	**P BH[b)]**	**Before lTRE**	**After lTRE**	**Change lTRE After – before (95% CI)**	**P[a)]**	**P BH[b)]**	**Difference between lTRE vs. eTRE**	**P[a)]**	**P BH[b)]**
PC_db_4	17.5	19.1	1.6 (-2.2 to -1.1)	**0.000006**	**0.00022**	17.8	18.7	0.93 (-1.3 to -0.42)	**0.00045**	**0.011**	-0.66 (-0.097 to 1.4)	0.08	0.34
PI_db_4	61.3	65	3.7 (-5.3 to -2.6)	**0.000008**	**0.00022**	61.7	63.8	2.1 (-3.3 to -0.92)	**0.0015**	**0.021**	-1.6 (-0.18 to 3.5)	0.07	0.33
PE_db_3	7.4	6.4	-0.98 (0.65 to 1.4)	**0.0000092**	**0.00022**	7.1	6.8	-0.28 (-0.16 to 0.7)	0.27	0.5	0.68 (-1.2 to -0.25)	**0.0099**	0.13
PC_db_6	6	6.7	0.76 (-1 to -0.38)	**0.000063**	**0.0011**	6	6.3	0.32 (-0.7 to 0.15)	0.23	0.46	-0.45 (-0.22 to 0.89)	0.17	0.47
CE_db_4	9.3	10.3	1 (-1.4 to -0.54)	**0.00012**	**0.0015**	9.4	10.3	0.88 (-1.2 to -0.48)	**0.00012**	**0.0042**	-0.12 (-0.49 to 0.73)	0.69	0.78
LPE_db_4	11.9	13.3	1.5 (-1.8 to -0.63)	**0.00012**	**0.0015**	12.3	12.2	-0.013 (-0.56 to 0.51)	0.87	0.92	-1.3 (0.42 to 2)	**0.0013**	**0.045**
CE_db_3	3.4	2.9	-0.45 (0.2 to 0.66)	**0.00023**	**0.0023**	3.4	3.2	-0.23 (0.0021 to 0.4)	**0.043**	0.23	0.21 (-0.52 to 0.14)	0.24	0.52
PC_db_3	10.5	9.8	-0.77 (0.33 to 1.2)	**0.00034**	**0.0027**	10.4	9.9	-0.5 (0.14 to 0.8)	**0.0071**	0.056	0.25 (-0.83 to 0.26)	0.3	0.53
PI_db_1	10.5	9.1	-1.4 (0.6 to 2.1)	**0.00038**	**0.0027**	10.5	9.9	-0.52 (-0.19 to 1.3)	0.16	0.4	0.92 (-2 to 0.043)	0.058	0.33
LPE_db_2	21.1	18.7	-2.4 (1.2 to 3.6)	**0.00042**	**0.0027**	20.7	20	-0.63 (-0.43 to 1.7)	0.36	0.58	1.8 (-3.4 to -0.018)	0.045	0.33
PI_db_3	8.9	8	-0.9 (0.46 to 1.4)	**0.00042**	**0.0027**	9	8.4	-0.59 (0.28 to 0.95)	**0.0012**	**0.021**	0.28 (-0.9 to 0.34)	0.43	0.54
Cer_db_2	30.7	32.8	2.1 (-2.9 to -0.72)	**0.00055**	**0.0031**	31.3	31.7	0.43 (-1.5 to 0.61)	0.4	0.6	-1.7 (0.43 to 3)	0.014	0.14
PE O-_db_5	47.2	50	2.8 (-4.1 to -0.99)	**0.00061**	**0.0031**	48.8	49.4	0.67 (-2.8 to 1.4)	0.56	0.7	-2.1 (-0.86 to 4.8)	0.21	0.49
SM_db_2	35.5	36.3	0.84 (-1.2 to -0.35)	**0.00061**	**0.0031**	35.6	36.2	0.56 (-0.94 to -0.22)	**0.0051**	0.052	-0.31 (-0.42 to 0.98)	0.35	0.53

PE_db_6	13.5	16.4	2.9 (-4.2 to -1.6)	**0.00074**	**0.0031**	13.8	14.6	0.78 (-2.7 to 0.93)	0.41	0.6	-1.8 (-0.66 to 4.3)	0.18	0.47
TAG_db_0	0.83	0.48	-0.34 (0.12 to 0.39)	**0.00075**	**0.0031**	0.84	0.75	-0.085 (-0.019 to 0.29)	0.095	0.29	0.15 (-0.36 to 0.2)	0.6	0.7
LPE_db_0	45.9	48.9	3 (-3.7 top -0.96)	**0.0008**	**0.0031**	46.7	46.3	-0.36 (-0.85 to 1.8)	0.49	0.68	-3.3 (0.78 to 4.4)	**0.0058**	0.13
PE_db_2	29.5	27.1	-2.4 (1 to 3.8)	**0.0008**	**0.0031**	29.9	28.2	-1.7 (0.22 to 2.5)	**0.022**	0.14	0.66 (-2.6 to 1.1)	0.31	0.53
LPC_db_0	65.2	67.7	2.6 (-3.7 to -0.89)	**0.00087**	**0.0033**	66.3	65.1	-1.2 (-0.11 to 2.2)	0.076	0.26	-3.8 (1.7 to 5.4)	**0.00014**	**0.0098**
SM_db_1	63.3	62.5	-0.77 (0.33 to 1.2)	**0.0011**	**0.004**	63.2	62.6	-0.58 (0.22 to 0.96)	**0.002**	**0.024**	0.23 (-0.93 to 0.45)	0.43	0.54
CE_db_6	0.89	0.99	0.096 (-0.14 to -0.041)	**0.0012**	**0.0041**	0.9	0.95	0.053 (-0.11 to 0.00064)	0.055	0.23	-0.04 (-0.035 to 0.087)	0.28	0.53
LPC_db_1	14	13.2	-0.87 (0.34 to 1.4)	**0.0013**	**0.0041**	13.6	13.8	0.28 (-0.59 to 0.33)	0.64	0.75	1.2 (-1.9 to -0.3)	**0.011**	0.13
PI_db_2	15	13.6	-1.3 (0.72 to 2.2)	**0.0013**	**0.0041**	14.5	13.6	-0.93 (-0.022 to 1.7)	0.058	0.23	0.44 (-1.7 to 0.82)	0.42	0.54
LPE_db_1	12	10	-1.9 (0.64 to 3)	**0.002**	**0.0057**	11.5	12.2	0.67 (-1.7 to 0.5)	0.25	0.46	2.5 (-4.5 to -0.69)	**0.016**	0.14
PC O-_db_2	14.4	13.2	-1.2 (0.31 to 1.8)	**0.002**	**0.0057**	14	14	-0.019 (-0.6 to 0.88)	0.57	0.7	1.2 (-1.9 to 0.039)	0.07	0.33
LPC_db_2	16.3	14.7	-1.6 (0.47 to 2.3)	**0.0024**	**0.0065**	15.6	16.2	0.52 (-1.2 to 0.5)	0.41	0.6	2.1 (-3.5 to -0.41)	**0.0099**	0.13
PC O-_db_3	12.5	11.6	-0.85 (0.25 to 1.3)	**0.003**	**0.0079**	12.3	11.9	-0.42 (-0.016 to 0.8)	0.061	0.23	0.46 (-1.1 to 0.21)	0.18	0.47
PE O-_db_3	9.5	8.5	-1 (0.3 to 1.4)	**0.0043**	**0.011**	9.1	8.6	-0.51 (-0.18 to 1)	0.25	0.46	0.47 (-1.4 to 0.63)	0.33	0.53
PC O-_db_5	24	25.4	1.4 (-2.5 to -0.48)	**0.0058**	**0.014**	23.8	24.6	0.79 (-1.9 to 0.3)	0.1	0.3	-0.61 (-0.98 to 2.4)	0.45	0.56
PE O-_db_4	6.4	5.8	-0.65 (0.18 to 1)	**0.0065**	**0.015**	6.3	6.1	-0.16 (-0.47 to 0.75)	0.58	0.7	0.55 (-1.4 to 0.24)	0.21	0.49
TAG_db_3	29.4	30.9	1.4 (-2.4 to -0.35)	**0.0076**	**0.017**	29.1	29.6	0.53 (-1.2 to 0.16)	0.11	0.32	-0.93 (-0.58 to 2.2)	0.29	0.53

LPC_db_4	3.5	3.8	0.24 (-0.44 to -0.051)	**0.012**	**0.026**	3.5	3.9	0.4 (-0.54 to -0.2)	**0.0000092**	**0.00065**	0.12 (-0.36 to 0.15)	0.34	0.53
LPE_db_6	9.2	10.2	1 (-1.7 to -0.2)	**0.013**	**0.028**	8.9	9.2	0.34 (-1.1 to 0.56)	0.53	0.7	-0.75 (-0.55 to 1.9)	0.38	0.54
PC_db_2	42.1	40.7	-1.3 (0.37 to 2.5)	**0.015**	**0.029**	41.9	40.9	-1 (0.006 to 1.6)	**0.046**	0.23	0.27 (-1.8 to 1.3)	0.97	0.97
PE_db_4	31.2	33	1.8 (-3.2 to -0.42)	**0.015**	**0.029**	31.7	32.4	0.62 (-2.6 to 0.72)	0.22	0.44	-1.1 (-0.96 to 3.4)	0.22	0.49
DAG_db_4	6.4	5.4	-1 (0.17 to 1.7)	**0.016**	**0.032**	6.1	6.8	0.7 (-1.5 to 0.72)	0.95	0.98	1.6 (-2.7 to 0.25)	0.097	0.36
Cer_db_1	63.1	61.7	-1.4 (0.18 to 2.6)	**0.017**	**0.033**	62.4	62.2	-0.2 (-0.47 to 1.4)	0.29	0.51	1.2 (-2.8 to 0.65)	0.2	0.48
TAG_db_1	9.5	8.1	-1.4 (0.22 to 2.3)	**0.017**	**0.033**	9.9	9	-0.9 (-0.069 to 1.8)	0.063	0.23	0.49 (-1.9 to 1.4)	0.86	0.88
PE O-_db_2	3.7	3.4	-0.38 (0.032 to 0.55)	**0.025**	**0.045**	3.6	3.4	-0.23 (0.0047 to 0.48)	0.05	0.23	0.18 (-0.63 to 0.31)	0.43	0.54
Cer_db_0	6.6	6.1	-0.51 (0.036 to 1.6)	**0.039**	0.07	6.7	6.7	-0.016 (-0.86 to 0.9)	0.97	0.99	0.5 (-1.9 to 0.4)	0.14	0.46
SM_db_0	1.2	1.1	-0.063 (0.0031 to 0.12)	**0.043**	0.074	1.2	1.2	0.02 (-0.096 to 0.032)	0.32	0.54	0.083 (-0.16 to -0.00029)	0.05	0.33
CE_db_2	56.3	55.9	-0.32 (-0.81 to 1.2)	0.67	0.7	55.9	54.7	-1.2 (0.37 to 2)	**0.0066**	0.056	-0.9 (-0.56 to 2.5)	0.18	0.47
TAG_db_9	0.41	0.32	-0.094 (-0.015 to 0.15)	0.11	0.18	0.29	0.4	0.11 (-0.21 to -0.027)	**0.014**	0.099	0.16 (-0.29 to 0.005)	0.074	0.33

Data are shown as mean or mean (95% CI).

Lipid classes & total double bonds per class are shown as, for example, “PC_db_4” meaning all measured phosphatidylcholines with 4 double bonds.

[a)] P-values without correction for multiple testing.

[b)] P-values with correction for multiple testing.

Table S13. Genes which mRNA expression was analyzed by the real-time PCR and primer sequences.

Gene symbol	Gene ID	Full name	Role	Circadian (tissue)	Forward primer	Reverse primer
FADS1/D5D	3998	Fatty acid desaturase 1	Desaturase	Yes[1] (liver)	5´-TGCCTTCAATGACTGGTTCA-3´	5´- ATGCTTGGCACACAAGGACT-3´
FADS2/D6D	9415	Fatty acid desaturase 2	Desaturase	Yes[1] (liver)	5´-AAGGGTGCCTCTGCCAACT-3´	5´-GATTGTAGGGCAGGTATTTCAGC-3´
SCD1/D9D	6319	Stearoyl-CoA desaturase	Desaturase	Yes[2] (liver)	5´-CATAACAGCAGGAGCTCATCGT-3´	5´-ACGAGCCCATTCATAGACATCA-3´
ELOVL5	60481	ELOVL fatty acid elongase 5	Elongase	Yes[1] (liver)	5´-TAACAGGAGTATGGGAAGGCA-3´	5´-ACGAGCCCATTCATAGACATCA-3´
ELOVL6	79071	ELOVL fatty acid elongase 6	Elongase	Yes[3] (liver)	5´-AACGAGCAAAGTTTGAACTGAGG-3´	5´-TCGAAGAGCACCGAATATACTGA-3´
FASN	2194	Fatty acid synthase	FA synthesis	Yes[4, 5] (adipose tissue and liver)	5´-AGACACTCGTGGCCTACAGCAT-3´	5´-ATGGCCTGGTAGGCGTTCT-3´
ACACA/ACC1	31	Acetyl-CoA carboxylase alpha	FA synthesis	Yes[4, 5] (adipose tissue and liver)	5´-TCGCTTTGGGGGAAATAAAGTG-3´	5´-TCGCTTTGGGGGAAATAAAGTG-3´,

ACSL1	2180	Acyl-CoA synthetase long chain family member 1	FA synthesis	Yes[6] (adipose tissue)	5´-AACAGACGGAAGCCCAAGC-3’	5´-TCGGTGAGTGACCATTGCTC-3´
CHPT1	56994	Choline phosphotransferase 1	Biosynthesis of PC	ye [7] (muscle)	5’-TCCAGTTCTTGGATTTCTAGGTGGAGT-3’	5´-ACACTGGTGCCTGCTATAGTGGA-3´
CEPT1	10390	Choline/ethanolamine phosphotransferase 1	Biosynthesis of PC	yes [7] (muscle)	5´-CAGTGATTGGAGGACCACCT-3´	5´-AGGACACTTGTTCCTGCTATTGT-3´
SPTLC2	9517	Serine palmitoyltransferase long chain base subunit 2	Biosynthesis of Cer	yes[7] (muscle)	5´-GAGACGCCTGAAAGAGATGG-3´	5´-TGGTATGAGCTGCTGACAGG-3´
CERS6	253782	Ceramide synthase 6	Biosynthesis of Cer	No[8] (estimated activity, serum)	5´-CGGACCTGAAGAACACGGAGGA-3´	5´-ATGGCGCACGGTTTGGCTAC-3´
GUSB	2990	Glucuronidase beta	Housekeeper		5´-CTCATTTGGAATTTTGCCGATT-3´	5´-CCGAGTGAAGATCCCCTTTTTA-3´
RPLP0	6175	Ribosomal protein lateral stalk subunit P0	Housekeeper		5´-GCTTCCTGGAGGGTGTCC-3´	5´-GGACTCGTTTGTACCCGTTG-3´

Supplemental References to the Table S13.

1. Chen, M., Y. Lin, Y. Dang, Y. Xiao, F. Zhang, G. Sun, X. Jiang, L. Zhang, J. Du, S. Duan, *et al.* "Reprogramming of rhythmic liver metabolism by intestinal clock." *J Hepatol* 79 (2023): 741-57. 10.1016/j.jhep.2023.04.040.
2. Zhou, X., D. Wan, Y. Zhang, Y. Zhang, C. Long, S. Chen, L. He, B. Tan, X. Wu and Y. Yin. "Diurnal variations in polyunsaturated fatty acid contents and expression of genes involved in their de novo synthesis in pigs." *Biochem Biophys Res Commun* 483 (2017): 430-34. 10.1016/j.bbrc.2016.12.126.
3. Chou, C. F., X. Zhu, Y. Y. Lin, K. L. Gamble, W. T. Garvey and C. Y. Chen. "Ksrp is critical in governing hepatic lipid metabolism through controlling per2 expression." *J Lipid Res* 56 (2015): 227-40. 10.1194/jlr.M050724.
4. Kohsaka, A., A. D. Laposky, K. M. Ramsey, C. Estrada, C. Joshu, Y. Kobayashi, F. W. Turek and J. Bass. "High-fat diet disrupts behavioral and molecular circadian rhythms in mice." *Cell Metab* 6 (2007): 414-21. 10.1016/j.cmet.2007.09.006.
5. Kudo, T., T. Tamagawa, M. Kawashima, N. Mito and S. Shibata. "Attenuating effect of clock mutation on triglyceride contents in the icr mouse liver under a high-fat diet." *J Biol Rhythms* 22 (2007): 312-23. 10.1177/0748730407302625.
6. Stenvers, D. J., A. Jongejan, S. Atiqi, J. P. Vreijling, E. J. Limonard, E. Endert, F. Baas, P. D. Moerland, E. Fliers, A. Kalsbeek, *et al.* "Diurnal rhythms in the white adipose tissue transcriptome are disturbed in obese individuals with type 2 diabetes compared with lean control individuals." *Diabetologia* 62 (2019): 704-16. 10.1007/s00125-019-4813-5.
7. Loizides-Mangold, U., L. Perrin, B. Vandereycken, J. A. Betts, J. P. Walhin, I. Templeman, S. Chanon, B. D. Weger, C. Durand, M. Robert, *et al.* "Lipidomics reveals diurnal lipid oscillations in human skeletal muscle persisting in cellular myotubes cultured in vitro." *Proc Natl Acad Sci U S A* 114 (2017): E8565-e74. 10.1073/pnas.1705821114.
8. Poolman, T. M., J. Gibbs, A. L. Walker, S. Dickson, L. Farrell, J. Hensman, A. C. Kendall, R. Maidstone, S. Warwood, A. Loudon, *et al.* "Rheumatoid arthritis reprograms circadian output pathways." *Arthritis Res Ther* 21 (2019): 47. 10.1186/s13075-019-1825-y.
9. Peters, B., D. A. Koppold-Liebscher, B. Schuppelius, N. Steckhan, A. F. H. Pfeiffer, A. Kramer, A. Michalsen and O. Pivovarova-Ramich. "Effects of early vs. Late time-restricted eating on cardiometabolic health, inflammation, and sleep in overweight and obese women: A study protocol for the chronofast trial." *Front Nutr* 8 (2021): 765543. 10.3389/fnut.2021.765543. https://www.ncbi.nlm.nih.gov/pubmed/34869534.
10. Peters, B., J. Schwarz, B. Schuppelius, A. Ottawa, D. A. Koppold, D. Weber, N. Steckhan, K. Mai, T. Grune, A. F. H. Pfeiffer, *et al.* "Effects of isocaloric early vs. Late time-restricted eating on insulin sensitivity, cardiometabolic health, and internal circadian time in women with overweight or obesity." *medRxiv* (2024): 2024.10.05.24314120. 10.1101/2024.10.05.24314120. https://www.medrxiv.org/content/medrxiv/early/2024/10/07/2024.10.05.24314120.full.pdf.

Table S14. Lipid pathway enrichment analysis of the plasma lipidome changes within eTRE intervention.

Pathway name	Pathway lipids	P-value[a)]	P-value[b)]	Signif.
Glycerophospholipid metabolism	26	0.0000137	0.00041	***
Sphingolipid signaling pathway	9	0.0032	0.0290	N.S.
Glycosylphosphatidylinositol (GPI)-anchor biosynthesis	3	0.0039	0.0290	N.S.
Autophagy - other	3	0.0039	0.0290	N.S.
Ferroptosis	11	0.0060	0.0324	N.S.
Autophagy - animal	4	0.0076	0.0324	N.S.
Necroptosis	4	0.0076	0.0324	N.S.
Choline metabolism in cancer	5	0.012	0.046	N.S.
Retrograde endocannabinoid signaling	8	0.032	0.104	N.S.
Pathogenic Escherichia coli infection	1	0.037	0.104	N.S.
Sphingolipid metabolism	21	0.038	0.104	N.S.
AGE-RAGE signaling pathway in diabetic complications	2	0.073	0.183	N.S.
Neurotrophin signaling pathway	3	0.108	0.216	N.S.
Adipocytokine signaling pathway	3	0.108	0.216	N.S.
Kaposi's sarcoma-associated herpesvirus infection	3	0.108	0.216	N.S.
Ether lipid metabolism	16	0.116	0.217	N.S.
Insulin resistance	4	0.141	0.235	N.S.
Leishmaniasis	4	0.141	0.235	N.S.
Tuberculosis	5	0.173	0.274	N.S.
Fat digestion and absorption	8	0.263	0.376	N.S.
Cholesterol metabolism	8	0.263	0.376	N.S.
Inositol phosphate metabolism	9	0.291	0.397	N.S.
Phosphatidylinositol signaling system	11	0.344	0.449	N.S.
Vitamin digestion and absorption	15	0.438	0.548	N.S.
Ovarian steroidogenesis	18	0.501	0.601	N.S.
alpha-Linolenic acid metabolism	23	0.590	0.666	N.S.
Linoleic acid metabolism	25	0.621	0.666	N.S.

Bile secretion	25	0.621	0.666	N.S.
Steroid biosynthesis	41	0.802	0.829	N.S.
Arachidonic acid metabolism	75	0.953	0.953	N.S.

Lipid pathway enrichment analysis was conducted using LIPEA software as described in *Experimental Section/Methods*.

[a)] P-values without correction for multiple testing.

[b)] P-values with Bonferroni correction for multiple testing.

Table S15. Key resources table.

Reagent or Resource	*Source*	*Identifier*
**Biological samples**		
Plasma and adipose tissue samples from women with overweight or obesity (the ChronoFast cohort)	German Institute of Human Nutrition Potsdam-Rehbruecke [9, 10]	Clinicaltrial.gov Identifier: NCT04351672
**Chemicals, peptides, and recombinant proteins**		
RNase-free Water	MP Biomedicals Ilc.	Cat.Nr: 7732-18-5
High-Capacity cDNA Reverse Transcription Kit	ThermoFisher Scientific / Applied Biosystems™	Cat.Nr: 4368814
RNase Inhibitor	ThermoFisher Scientific / Applied Biosystems™	Cat.Nr: N8080119
384-Well Multiply®-PCR Plate	ThermoFisher Scientific / Applied Biosystems™	Cat.Nr: AB1384
*Power*SYBR® Green PCR Master Mix	ThermoFisher Scientific / Applied Biosystems™	Cat.Nr: 4367659
qPCR Primers	Invitrogen	N/A
**Deposited data**		
Raw RNAseq data for adipose tissue samples	Gene Expression Omnibus (GEO) database https://www.ncbi.nlm.nih.gov/geo/	GSE287198
Plasma lipidomics data	Figshare database	https://doi.org/10.6084/m9.figshare.30172450.v2
Lipidomics Standard Initiative minimal reporting checklist	Zenodo database	https://doi.org/10.5281/zenodo.15183498
**Software and algorithms**		
MinimPy	Open source software	https://sourceforge.net/projects/minimpy/
SPSS Statistics 25.0	IBM	https://www.ibm.com/de-de/products/spss-statistics
GraphPad Prism Version 5.0	GraphPad	https://www.graphpad.com/

Bodygram™	AKERN	https://www.akern.com/en/products-and-solutions/data-analysis-software/bodygram-software/
Fddb App and Food Database	Food Database GmbH	https://fddb.info/
ActiLife Version 6.13.4	ActiGraph	https://theactigraph.com/
QuantStudio™ Real-Time PCR Software	ThermoFisher Scientific	https://www.thermofisher.com/de/de/home/global/forms/life-science/quantstudio-6-7-flex-software.html
FastQC v0.12.1	Open source http://www.bioinformatics.babraham.ac.uk/projects/fastqc/	RRID:SCR_014583
STAR v2.7.11a	Open source https://github.com/alexdobin/STAR	RRID:SCR_004463
STRINGTIE v2.2.1	Open source https://ccb.jhu.edu/software/stringtie/	RRID:SCR_016323
DESeq2 v1.34.0	Open source https://bioconductor.org/packages/release/bioc/html/DESeq2.html	RRID:SCR_015687
Python v3.10.9	Open source https://www.python.org/	RRID:SCR_008394
R v4.1.2	Open source https://www.r-project.org/	RRID:SCR_001905
LIPEA	Biomedical Cybernetics Group https://hyperlipea.org/	N/A

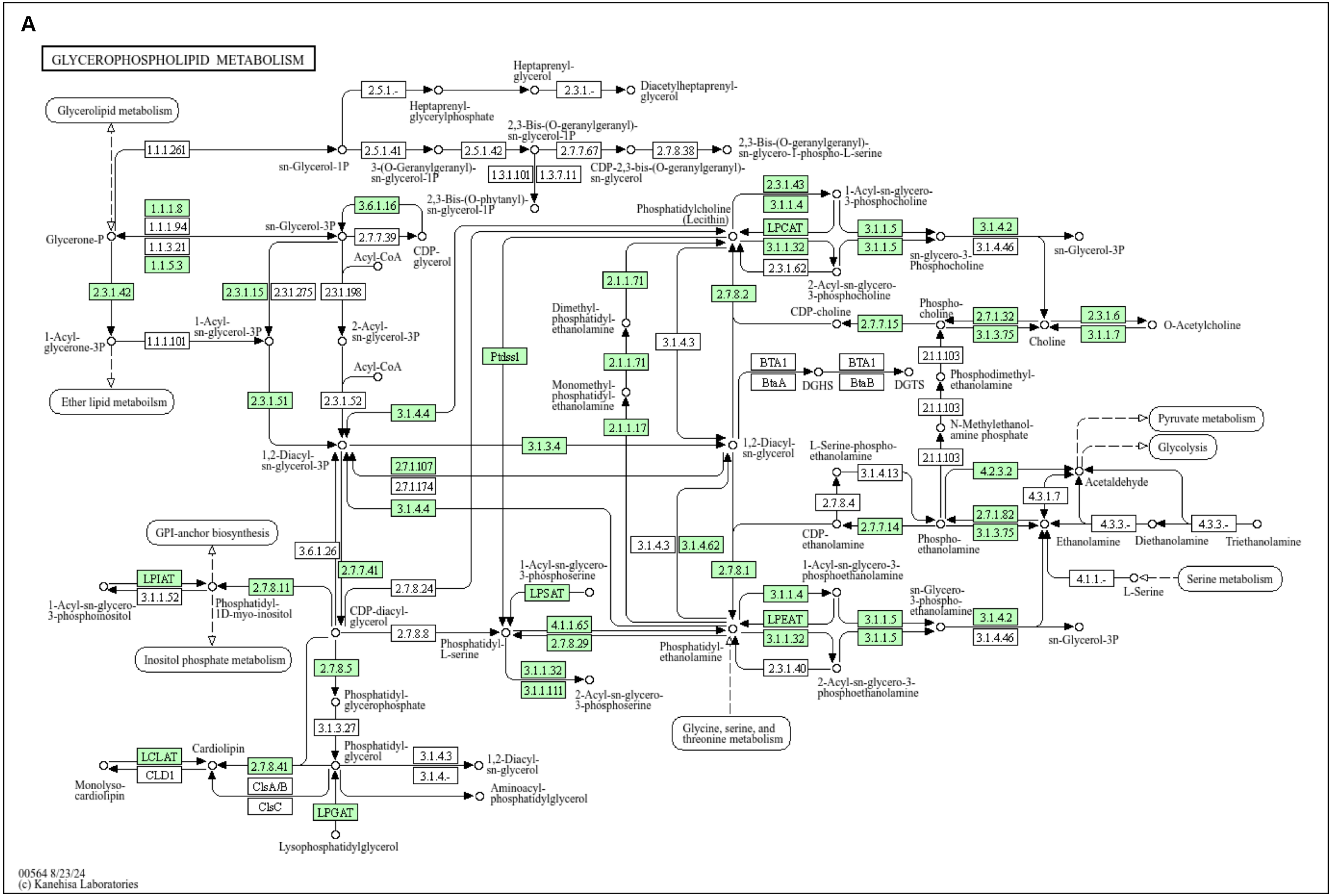
A
GLYCEROPHOSPHOLIPID METABOLISM
Glycerolipid metabolism
Ether lipid metaboilsm
GPI-anchor biosynthesis
Inositol phosphate metabolism
Pyruvate metabolism
Glycolysis
Serine metabolism
Glycine, serine, and threonine metabolism
Glycerone-P
sn-Glycerol-1P
sn-Glycerol-3P
1-Acyl-glycerone-3P
1-Acyl-sn-glycerol-3P
2-Acyl-sn-glycerol-3P
1,2-Diacyl-sn-glycerol-3P
CDP-diacyl-glycerol
Phosphatidyl-L-serine
Phosphatidyl-ethanolamine
Phosphatidylcholine (Lecithin)
1,2-Diacyl-sn-glycerol
Cardiolipin
Monolyso-cardiolipin
Phosphatidyl-glycerophosphate
Phosphatidyl-glycerol
Lysophosphatidylglycerol
Aminoacyl-phosphatidylglycerol
Choline
O-Acetylcholine
Ethanolamine
Diethanolamine
Triethanolamine
L-Serine
Acetaldehyde
00564 8/23/24
(c) Kanehisa Laboratories

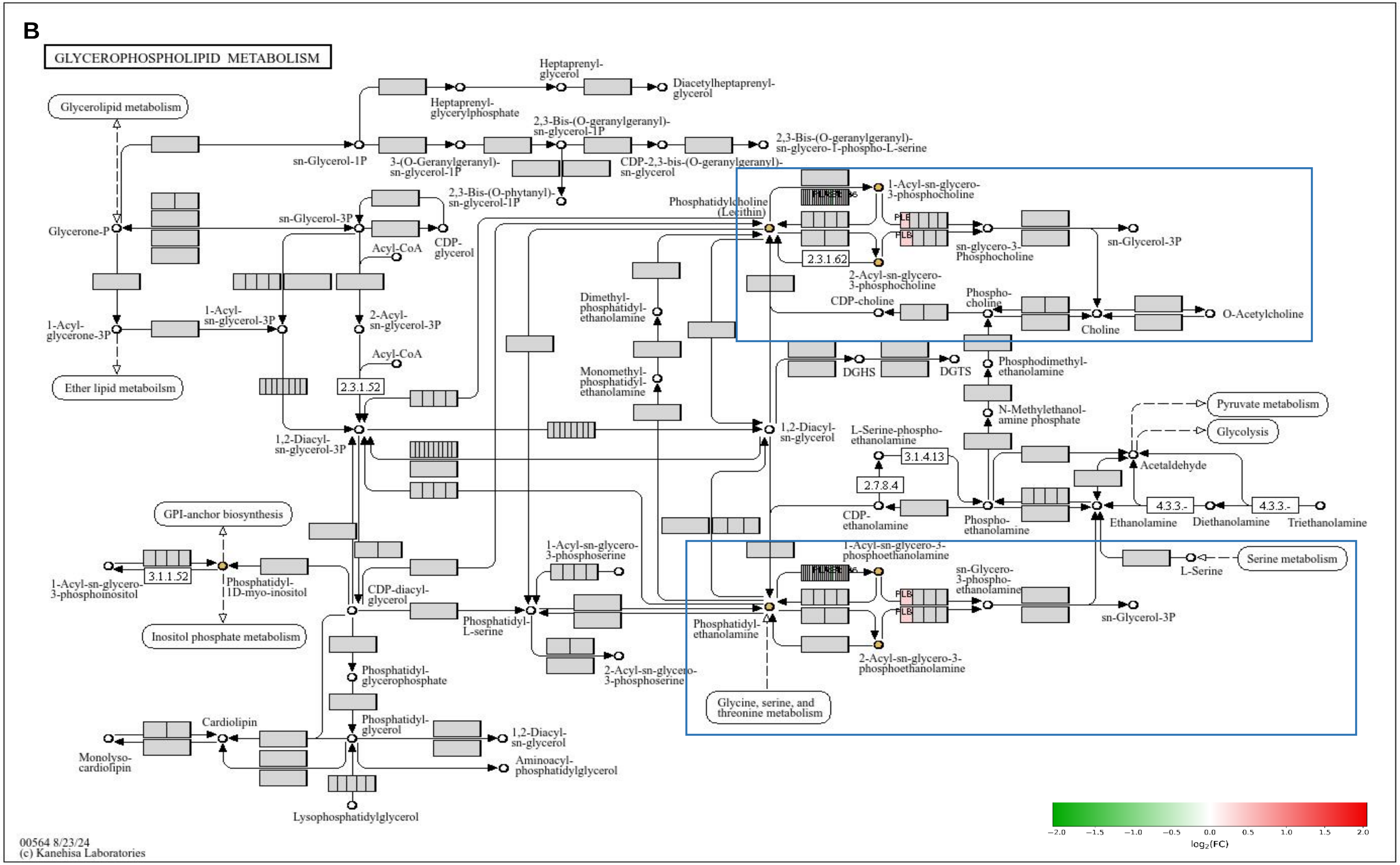
B
GLYCEROPHOSPHOLIPID METABOLISM
Glycerolipid metabolism
Ether lipid metaboilsm
GPI-anchor biosynthesis
Inositol phosphate metabolism
Glycine, serine, and threonine metabolism
Pyruvate metabolism
Glycolysis
Serine metabolism
Heptaprenyl-glycerol
Diacetylheptaprenyl-glycerol
Heptaprenyl-glycerylphosphate
2,3-Bis-(O-geranylgeranyl)-sn-glycerol-1P
2,3-Bis-(O-geranylgeranyl)-sn-glycero-1-phospho-L-serine
sn-Glycerol-1P
3-(O-Geranylgeranyl)-sn-glycerol-1P
CDP-2,3-bis-(O-geranylgeranyl)-sn-glycerol
2,3-Bis-(O-phytanyl)-sn-glycerol-1P
Glycerone-P
sn-Glycerol-3P
Acyl-CoA
CDP-glycerol
1-Acyl-glycerone-3P
1-Acyl-sn-glycerol-3P
2-Acyl-sn-glycerol-3P
2.3.1.52
1,2-Diacyl-sn-glycerol-3P
Phosphatidylcholine (Lecithin)
1-Acyl-sn-glycero-3-phosphocholine
2.3.1.62
2-Acyl-sn-glycero-3-phosphocholine
sn-glycero-3-Phosphocholine
sn-Glycerol-3P
Phospho-choline
CDP-choline
Choline
O-Acetylcholine
Dimethyl-phosphatidyl-ethanolamine
Monomethyl-phosphatidyl-ethanolamine
DGHS
DGTS
Phosphodimethyl-ethanolamine
N-Methylethanol-amine phosphate
1,2-Diacyl-sn-glycerol
L-Serine-phospho-ethanolamine
3.1.4.13
2.7.8.4
Acetaldehyde
CDP-ethanolamine
Phospho-ethanolamine
Ethanolamine
4.3.3.-
Diethanolamine
Triethanolamine
L-Serine
1-Acyl-sn-glycero-3-phosphoserine
1-Acyl-sn-glycero-3-phosphoethanolamine
sn-Glycero-3-phospho-ethanolamine
sn-Glycerol-3P
Phosphatidyl-ethanolamine
2-Acyl-sn-glycero-3-phosphoethanolamine
Phosphatidyl-L-serine
2-Acyl-sn-glycero-3-phosphoserine
3.1.1.52
1-Acyl-sn-glycero-3-phosphoinositol
Phosphatidyl-1D-myo-inositol
CDP-diacyl-glycerol
Phosphatidyl-glycerophosphate
Phosphatidyl-glycerol
1,2-Diacyl-sn-glycerol
Aminoacyl-phosphatidylglycerol
Cardiolipin
Monolyso-cardiolipin
Lysophosphatidylglycerol
PLB
−2.0 −1.5 −1.0 −0.5 0.0 0.5 1.0 1.5 2.0
log2(FC)
00564 8/23/24
(c) Kanehisa Laboratories

Figure S1. Full map of the glycerophospholipid pathway combining lipid and gene expression data.

(A) Glycerophospholipid pathway identified by the lipid pathway enrichment analysis of plasma lipidome before and after the eTRE intervention. Pathway enzymes which activity were potentially altered by this intervention are highlighted in green.

(B) Glycerophospholipid pathway combining lipid and gene expression data. The output of the glycerophospholipid pathway revealed by the lipid pathway enrichment analysis was subjected to the metaKEGG tool together with the SAT transcriptome dataset. Genes found in the transcriptome dataset were directly mapped on the pathway, colored according to their log_2FC translated to a color scale, while KEGG compounds were assigned a single color to highlight their presence in the pathway. In the color scale, red color means a positive log2FC, i.e. the transcript was upregulated in ITRF compared to the eTRE, whereas green color means a negative log2FC. Areas zoomed in Figure3 are highlighted with a blue frame.
